# How best to capture the impact of complementary therapies in palliative care: A systematic review to identify and assess the appropriateness and validity of multi-domain tools

**DOI:** 10.1177/02692163221122955

**Published:** 2022-09-07

**Authors:** Lucy Mitchinson, Christina Chu, Andrea Bruun, Ali-Rose Sisk, Megan Armstrong, Cecilia Vindrola-Padros, Nuriye Kupeli, Bridget Candy, Patrick Stone

**Affiliations:** 1 Marie Curie Palliative Care Research Department, University College London, London, UK; 2 Primary Care and Population Health, University College London, London, UK; 3 Department of Targeted Intervention and Rapid Research, Evaluation and Appraisal Lab (RREAL), University College London, London, UK

**Keywords:** Systematic review, palliative care, complementary therapies, patient reported outcome measures, quality of life

## Abstract

**Background:**

Complementary therapies are widely used in palliative care settings. Qualitative research found that people with advanced disease report a range of physical and psychological benefits from complementary therapies, however evidence of their effectiveness from clinical trials is inconclusive. This may be because trials are limited by use of inappropriate outcome measures.

**Aims:**

To identify tools which capture the impact of massage, reflexology and aromatherapy in people with advanced disease. We (1) identified multi-domain tools used to evaluate these therapies in populations with any chronic health condition and (2) assessed whether tools were valid and psychometrically robust in populations with advanced disease.

**Design:**

A two-stage systematic review was conducted using the COnsensus-based Standards for the selection of health Measurement INstruments (COSMIN) guidelines (PROSPERO: CRD42020161199).

**Data sources:**

Six databases were searched (August 2021). Study methodological quality, tool psychometric properties and evidence quality were assessed. A global comparison score was generated.

**Results:**

Stage 1: 66 trials using 40 different multi-domain tools were identified. Stage 2: Of these tools, we identified papers for seven tools regarding development or validation in advanced disease populations. The majority of psychometric data were inconsistent or inconclusive. Data were mostly of low quality due to methodological issues.

**Conclusion:**

Of the tools identified, ‘Functional Assessment of Cancer Therapy – General’ appears to be the most suitable alternative tool against COMSIN criteria, for trials of massage, reflexology and aromatherapy in palliative care. Further tool validation is required before firm recommendations can be made. Co-development of a core outcome set could ensure relevant domains are assessed.

**What is already known about the topic?**

- Complementary therapies are popular with people with advanced disease.
- Trial evidence for the effectiveness of massage, reflexology and aromatherapy style therapies in this population is inconclusive.
- It is unknown whether the inconclusive findings are a reflection of limitations of previously used outcome measures.

**What this paper adds?**

- The validation of multi-domain outcome measurement tools in populations with advanced disease is limited.
- Of the seven multi-domain tools identified, the Functional Assessment of Cancer Therapy – General (FACT-G) score was deemed to be the most suitable as it had the greatest evidence for sufficient psychometric properties.

**Implications for practice, theory or policy?**

- As current validation is limited, further research is needed to develop tools and validate psychometric properties of existing tools for populations with advanced disease.
- Establishing which tools are most appropriate for use will facilitate the standardisation of outcome measurement across trials and aid cross-study comparisons of complementary therapy effectiveness.
- Improving how the impact of complementary therapies is measured may provide support for their continued use in palliative care settings.

## Introduction

People with advanced disease commonly face physical, emotional and spiritual distress. Palliative care therefore takes a broad and holistic approach to support patients and their families.^1^ In recent years there has been an increase in use of alternative and complementary therapies by general public and clinical populations.^2,3^ Complementary therapies can be used alongside traditional medicine to help people cope with their illness, with some of the most popular being aromatherapy and touch-based therapies such as massage or reflexology.^4^

However, despite greater demand for these therapies in care settings, clinical trial evidence on their impact in palliative care remains inconclusive. A recent review of randomised control trials (RCTs) of some of the most popular therapies (massage, reflexology and aromatherapy) in advanced disease populations, found that trials had low methodological quality and reported inconsistent effects.^5^ While these findings are unfavourable to complementary therapies, qualitative research provides a different picture. A review of qualitative studies on the impact of massage, aromatherapy and reflexology found that people with advanced disease report a range of benefits addressing social, emotional, physical and spiritual needs.^6^

The reason for the disconnect between quantitative and qualitative findings is unclear. Measuring quantitative outcomes in palliative care can be a challenge as patients are deteriorating and studies are commonly underpowered due to small samples.^7^ A previous trial demonstrated how intervention effects could be overlooked; when a reduction in rate of decline was identified rather than a significant improvement in patients.^8^ A mixed-methods synthesis review suggests clinical trials use tools which do not capture the full range of domains which are important to patients, in part explaining why complementary therapies show little to no benefit.^9^ Developing a core outcome set (a list of outcomes recommended by key stakeholders to be measured in all future trials of a specific topic) may be necessary to ensure meaningful outcomes are evaluated. Alternatively, inconsistent findings on effectiveness may also be in part due to the use of outcome measurement tools which have not been developed or validated in this population. Despite the growing number of available tools, psychometric quality is not always adequately examined in populations with advanced disease.^10,11^

These methodological challenges make it difficult to determine the true effect of complementary therapies. While further work is required to understand which domains are of most importance to patients, we need to identify existing alternatives so research into effectiveness can continue. Clear guidance is needed to ensure tool selection is appropriate for the assessment of complementary therapies, and valid for use with people with advanced disease. Tools used to evaluate complementary therapies with other clinical populations may begin to capture therapy specific benefits and therefore be suitable alternatives. Ensuring these identified tools are psychometrically sound may offer a starting point for addressing these methodological challenges. Tools could be altered to address other identified domains and be a suitable alternative for exploring the effects of complementary therapies on patients in palliative care.

In this paper, we sought to identify multi-domain tools which may be appropriate outcome measures for evaluating massage, reflexology and aromatherapy complementary therapies in people living with advanced disease. Our objectives were:

1. To identify multi-domain tools used to evaluate massage, reflexology and aromatherapy in RCTs in clinical populations with chronic health conditions (beyond palliative care).
2. To determine which multi-domain tools may be most suitable for use in palliative care by evaluating the psychometric properties of those tools which were developed or validated in populations with advanced disease.

## Methods

COnsensus-based Standards for the selection of health Measurement INstruments (COSMIN) guidelines for systematic reviews of Patient Reported Outcome Measures (PROMs) were followed.^12^ The protocol was registered on PROSPERO (CRD42020161199; https://www.crd.york.ac.uk/prospero/display_record.php?RecordID=161199) (see Supplemental Material for adaptions).^13^

### Search 1: Identify multi-domain tools used in RCTs of massage, reflexology and aromatherapy in clinical populations

A search containing variations on the terms ‘massage’, ‘reflexology’, ‘aromatherapy’ was run across EMBASE, MEDLINE, CINAHL, AMED and CENTRAL databases from inception to June 2021. Search restrictions were set for; English language, Humans, Adults and clinical trials (see Supplemental Material).

### Inclusion and exclusion criteria

RCTs which addressed the following criteria were included: (1) Population: adults (>18 years) with a diagnosed chronic physical or mental health condition. Studies involving people with; injury, pregnancy, childbirth, period pain, menopause, lacking mental capacity or undergoing surgery were excluded, as these populations were deemed too different to the population of interest. (2) Intervention: Any massage, reflexology or aromatherapy type intervention. Studies reporting on therapies focusing on a single physical outcome (e.g. physiotherapy-style treatments, therapies for specific symptoms or manual lymphatic drainage) or delivered within a complex intervention were excluded. (3) Comparison: Any form of comparator or control. (4) Outcome: Any outcome assessed using a multi-domain outcome tool. Multi-domain tools were defined as those which measure both physical symptoms and/or emotional, social or spiritual effects. Trials using trial-specific tools, visual analogue scales and proxy measures were excluded.

Trials could be from any country but must be peer reviewed and published in English. On-going trials, feasibility or pilot studies were excluded.

### Study selection and data extraction

One author (LM) screened all titles and abstracts against the criteria, other authors (BC, NK, A-R.S, AB, MA) independently screened one fifth each. Any discrepancies were discussed until consensus was reached. Full text of relevant citations were reviewed. Study aims, intervention, population, trial setting and outcome tools used were extracted. Processes undertaken by a single reviewer were checked. Single review was only permitted after consensus was reached. Tools identified as multi-domain were included in the following search.

### Search 2: Psychometric quality of tools developed or validated in advanced disease

A second search was conducted to determine which of the tools identified in *search 1* had been developed and/or validated in people living with advanced disease. The search included various terms for ‘advanced disease’, the names of identified tools and relevant terms recommended for this purpose^14^ (see Supplemental Material). The search was run on EMBASE, MEDLINE and PsychInfo from inception to August 2021, with limits set for English language and Human studies. Reference lists of studies included in *search 1* were also reviewed.

### Inclusion and exclusion criteria

Studies were included which met the following criteria. (1) Population: At least 50% of the population had to be at an advanced stage of disease (described as advanced, metastatic, or Stage 3 or 4) and be over 18 years of age. Studies where participants were carers, people with reduced mental capacity and survivors were excluded. (2) Intervention: Studies were included if they reported on development or validation of identified tools in people living with advanced physical disease. Literature reviews and case studies were excluded.

Papers reporting on non-English versions of the tool were excluded as potential differences in translation may have affected how benefits were perceived. Studies which used only sub-scales or single items from a larger tool were excluded. However, studies which included minor modifications to the original tool (such as different recall periods) were included. (3) Comparison: Studies with any form of comparator or no comparator were included. (4) Outcome: The outcome of interest was any qualitative or psychometric data relating to the development or quality of the identified tool.

### Study selection and data extraction

One author (LM) screened all titles and abstracts, and three authors (NK, A-R.S, AB) second-screened results. Any discrepancies were discussed to reach consensus. Full text articles were then re-screened against the criteria.

Key information was extracted from the full text articles; author(s), date, tool name, sample population, method of administration, recall period, number of items, response options, range of scores, tool characteristics, measurement properties, and interpretability and feasibility.

### Assessment of methodological quality

Each study was rated against items on the COSMIN Risk of Bias checklist.^15^ An overall rating of ‘very good’, ‘adequate’, ‘doubtful’ or ‘inadequate’ was assigned for each measurement property. Two authors (LM, CC) rated independently with discrepancies discussed with a third author (NK) to reach consensus.

### Assessment of psychometric properties

Psychometric property data reported in the study were rated against the ‘Good Measurement Properties’ criteria (see Supplemental Material).^12^ Two reviewers independently rated each property as either sufficient (+), insufficient (−) or indeterminate (?). Ratings were then compared and discussed to reach consensus.

Studies reporting on content validity or tool development were rated against the ‘Ten Criteria for Good Content Validity’ (see Supplemental Material). An overall rating of sufficient (+), insufficient (−), inconsistent (+/−) or indeterminate (?) was generated by combining reported data with the reviewer’s own assessment of tool content.

Studies using the same tool were assessed to determine whether data were similar enough to be summarised. When possible, summarised data were rated again to determine overall quality of the tool. When studies used different versions and modified tools (e.g. with alternative recall periods, response formats or response anchors) psychometric data could not be grouped. Data for these tools are presented separately. All evidence, regardless of quality rating, was included in the evaluation.

### Levels of evidence

The Grading of Recommendations Assessment, Development and Evaluation (GRADE) approach^16^ was used to determine trustworthiness of data for each outcome measurement scale. Described as either; High, Moderate, Low or Very-Low (Table 1), ratings were determined by risk of bias, inconsistency, indirectness of studies and imprecision (e.g. small sample size). In cases where an inconsistent rating was given a GRADE score could not be provided.

**Table 1.** Definition of GRADE evidence levels.^16^

Quality level	Definition
High	We are very confident that the true measurement property lies close to that of the estimate* of the measurement property
Moderate	We are moderately confident in the measurement property estimate: the true measurement property is likely to be close to the estimate of the measurement property, but there is a possibility that it is substantially different
Low	Our confidence in the measurement property is limited: the true measurement property may be substantially different from the estimate of the measurement property
Very low	We have very little confidence in the measurement property estimate: the true measurement property is likely to be substantially different from the estimate of the measurement property

* Estimate of the measurement property refers to the pooled or summarised result of the measurement property of the tool.

### Scoring system

To make global comparisons between tools, a scoring system developed by Kupeli et al.^17^ was applied (Table 2). The psychometric rating determined by the COSMIN ‘Good Measurement Properties’ criteria specified the direction of the score as positive or negative. Quality of evidence, as determined by GRADE, specified the magnitude of the score, with higher quality evidence receiving a greater number of points. Psychometric properties rated as indeterminate or inconsistent were scored as 0. Values were averaged per multi-domain tool to account for number of studies.

**Table 2.** Kupeli scoring system for global tool comparison.^17^

Psychometric rating	Level of evidence	Score assigned
+	High	+4
+	Moderate	+3
+	Low	+2
+	Very Low	+1
−	Very Low	−1
−	Low	−2
−	Moderate	−3
−	High	−4

COSMIN categories for tool recommendations were also applied.^12^ Tools with sufficient evidence for content validity and (at least low level) evidence for sufficient internal consistency, are categorised as Band A and recommended for use. Tools which require further research but have the potential to be recommended are categorised as Band B. Tools with high quality evidence for an insufficient measurement property are categorised as Band C and receive recommendations against their use.

## Results

**Figure 1.**
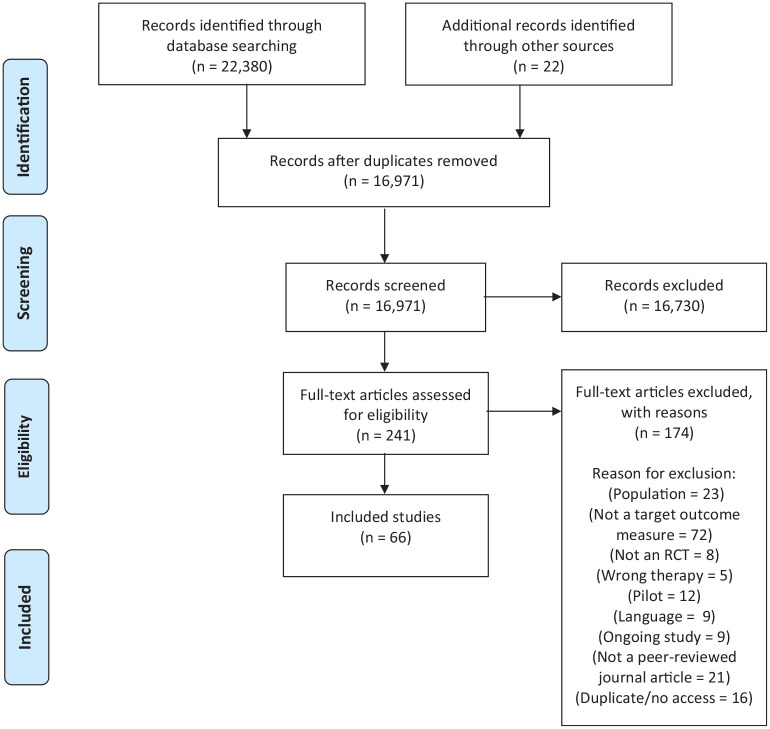
PRISMA flowchart search 1.

**Figure 2.**
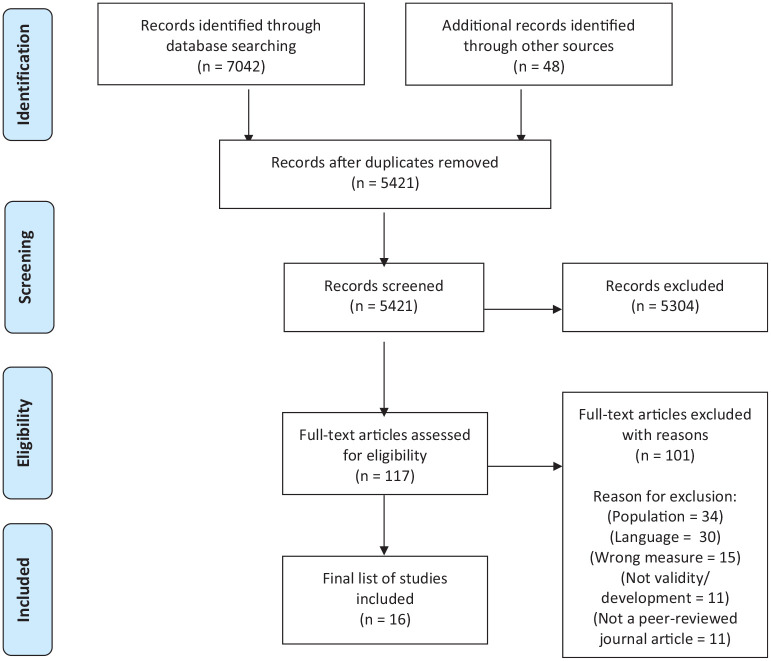
PRISMA flowchart search 2.

*Search 1* identified 16,971 citations. After screening titles and abstracts, 241 papers were reviewed at full text and 66 met inclusion criteria (references in Supplemental Material). From these RCTs, 40 multi-domain outcome tools were identified. *Search 2* identified 5421 studies for title and abstract screening. Of these, 117 met inclusion criteria and full text papers were screened. After exclusions were applied, 16 tool development and/or validation papers reporting on seven multi-domain tools in people living with advanced disease, were included. Figures 1 and 2 present the PRISMA flowcharts for *search 1* and *search 2* respectively.

### Study characteristics

Sample characteristics for both searches are displayed in Table 3. RCTs identified from *search 1* were conducted across 20 countries. Participants were recruited from both in-patient and out-patient facilities. Cancer was the most common diagnosis. Massage was the most common intervention (*n* = 19), followed by acupressure (a therapy similar to reflexology) (*n* = 15), reflexology (*n* = 13), aromatherapy-massage (*n* = 6), aromatherapy (*n* = 4), healing touch (*n* = 1), self-acupoint massage (*n* = 1), manual therapy (*n* = 1), massotherapy (*n* = 1), massage and aromatherapy (*n* = 2), healing touch and relaxation (*n* = 2) and massage and reflexology (*n* = 1). Trials used 40 different multi-domain tools which covered; quality of life (*n* = 19), mood (*n* = 5), symptoms (*n* = 4), health status (*n* = 3), wellbeing (*n* = 3), comfort (*n* = 2), functioning (*n* = 1), life satisfaction (*n* = 1) and psychiatric disorders (*n* = 1). The most commonly used (14/66 studies) tool was the Short-Form 36 Health survey (SF-36). (See extracted data in Supplemental Material).

**Table 3.** Sample demographics of Search 1 to Identify multi-domain tools used in RCTs of massage, reflexology, and aromatherapy in clinical populations, and Search 2 to determine which of the tools identified had been developed and/or validated in people living with advanced disease.

	Search 1	Search 2
Mean age (range)	12–94 years	43–70 years
% female (range)	0–100	3.43–79.5
Clinical condition (n)		
Cancer	22	13^*^
Haemodialysis	10	
Diabetics	5	
Cardiac issues/heart disease	3	
Palliative care	3	2
Migraine	2	
HIV/AIDS	2	1^*^
Asthma	2	
Osteoarthritis	2	
Fibromyalgia	2	
Multiple sclerosis	2	
Depression	1	
Epilepsy	1	
Hypertension	1	
Carpal tunnel	1	
Sickle cell pain	1	
Liver transplant	1	
Bone marrow transplant	1	
Stem cell transplant	1	
Functional disorders	1	
Chronic Brachial Neuralgia	1	
Tension type headaches	1	
Parkinsonian disorders		1
Recruited from (*n*)		
Hospital	35	6
Specialist clinics/centres	13	2
Palliative care/hospice	4	6
Medical centres	4	
Community care	4	
Societies and universities	3	
Both hospitals and medical centres	1	
Unclear	2	2
Country of study (*n*)		
United States of America	14	7^♦^
Turkey	10	
Iran	8	
China	7	
United Kingdom	4	4
Denmark	4	
Brazil	3	
Malaysia	3	
Germany	2	
Australia	1	1
Belgium	1	
Canada	1	3^∆ ♦^
India	1	
Israel	1	
Japan	1	
Korea	1	
Nigeria	1	
Spain	1	
Sweden	1	
Taiwan	1	
Netherlands		1
Singapore		1
Switzerland		1^∆^

* 1 study recruited people with either cancer or AIDs.

∆ 1 study recruited from Canada and United States of America.

♦ 1 study recruited from Canada and Switzerland.

Psychometric properties of tools

Studies in *search 2* were conducted in seven countries. Sample populations most commonly had advanced cancer and were recruited from hospitals or specialist palliative care settings. Characteristics of the seven multi-domain tools included are presented in Table 4. Tools assessed the symptom distress, psychological distress, quality of life, and comfort at the end-of-life. The identified tools were; Hospice Comfort Questionnaire, General Health Questionnaire-12 (GHQ-12), Functional Assessment of Cancer Therapy-General (FACT-G), European Organisation of Research and Treatment of Cancer Quality of life Questionnaire Core 30 (EORTC-QLQ C30), Rotterdam Symptom Checklist, Edmonton Symptom Assessment System (ESAS) and the McGill Quality of Life Questionnaire. Studies evaluated tools overall (*n* = 5), individual subscales (*n* = 4), or both overall tool and subscales (*n* = 7). Some studies compared target tool to other outcome measures (*n* = 9), other outcome measures and alternative versions of the tool (*n* = 3), specific aspects of tool development (*n* = 2), alternative versions of the tool (*n* = 1) or evaluated other outcome measures and specific aspects of tool development (*n* = 2).

**Table 4.** Details of identified tools.

Instrument	Year of develop.	Original Language	Construct; Domains	No. of items	Target Population	Response Options	Range of scores	Recall period	Mode of admin.
Hospice Comfort Questionnaire (version 1)	2001	English	End-of-Life Comfort; Physical, Psychospiritual, Environmental, Social.	49 (+1 optional)	Patients in hospice care	6-point scale; Strongly Agree – Strongly Disagree	49–294	Current	Self-report
Hospice Comfort Questionnaire (version 2)	*As above.*	*As above*	*As above*	*As above*	*As above*	4-point scale; Strongly Agree – Strongly Disagree	49–196	*As above*	*As above*
FACT-G	1993	English	Quality of Life; Physical Wellbeing, Social/family wellbeing, Emotional wellbeing, Functional wellbeing.	28	Cancer Patients (18+)	5-point scale; Not at all, A little bit, Somewhat, Quite a bit, Very much.	0–112	Past 7 days	Self-report
EORTC QLQ C30 Version 1	1993	English	Health Related Quality of Life; Global health status/quality of life, Physical functioning, Role functioning, Cognitive functioning, Emotional functioning, Social functioning. Fatigue, Pain, Nausea/vomiting, Dyspnoea, Loss of appetite, Insomnia, Constipation, Diarrhoea, Financial difficulties.	30	Cancer patients	First 7 items: Yes or no. Following 21 items: 4-point scale; Not at all A little Quite a bit Very much Following 2 items: 7-point scale; 1 Very Poor – 7 Excellent	0–105 or linear transformation for 0–100 scale.	During the past week	Self-report
EORTC QLQ C30 Version 2	1997	*As above*	*As above*	*As above*	*As above*	First 5 items: Yes or no. Following 23 items: 4-point scale; Not at all A little Quite a bit Very much Following 2 items: 7-point scale; 1 Very Poor – 7 Excellent	0–111 or linear transformation for 0–100 scale	*As above*	*As above*
EORTC QLQ C30 Version 3	2000	*As above*	*As above*	*As above*	*As above*	First 28 items: 4-point scale; Not at all A little Quite a bit Very much Following 2 items: 7-point scale; 1 Very Poor – 7 Excellent	0–126 or linear transformation for 0–100 scale	*As above*	*As above*
EORTC QLQ C30 Version 3 modified	*As above*	*As above*	*As above*	*As above*	*As above*	*As above*	*As above*	24-h	*As above*
GHQ-12	1980	English	Psychological Distress; 2 and 3 factor structures suggested.	12	General Population	4-point scale; Positive phrased items: Better than usual, Same as usual, Less than usual Much less than usual. 4-point scale; Negative phrased items: Not at all, No more than usual, Rather more than usual, Much more than usual.	12–48	Past few weeks	Self-report
Rotterdam Symptom Checklist	1990	Dutch	Symptom Distress; Physical, Psychological, Activities of daily living.	38	Cancer patients	4-point scale; Not at all, A little, Quite a bit, Very much. 4-point scale; Unable, Only with help, Without help with difficulty, With difficulty	38–152	Past 7 days	Self-report
ESAS Likert (best describes)	1991	English	Symptoms distress; Pain, Tiredness, Drowsiness, Nausea, Lack of Appetite, Shortness of Breath, Depression, Anxiety, Wellbeing, Other. Diagram to mark pain on body.	10	Palliative care	11-point scale; No [symptom] to Worst possible [symptom]	0–100	‘that best describes’:	Self-report
ESAS Likert modified (24-h)	*2000*	*As above*	*As above*	*As above*	*As above*	*As above*	*As above*	Symptoms over the last 24-h	*As above*
ESAS-revised	2012	*As above*	*As above*	*As above*	Advanced cancer patients	*As above*	*As above*	‘that best describes how you feel now’	*As above*
McGill Quality of Life (7-point scale)	1995	English	Quality of Life; Physical well-being, Physical symptoms, Psychological symptoms, Existential well-being, Support.	17	Advanced disease, Palliative care	1–7 numeric rating scale; No problem – tremendous problem	17–119	In the past 2 days	Self-report
McGill Quality of Life (11-point scale)	1997	*As above*	*As above*	*As above*	*As above*	11-point scale; No problem – tremendous problem Qualitative section: ‘Please list or describe the things which had the greatest effect on your quality of life in the past two days’.	0–160	*As above*	*As above*

FACT-G: Functional Assessment of Cancer Therapy – General; EORTC QLQ C30: European Organization for Research and Treatment of Cancer-Quality of Life-Core 30; GHQ-12: General Health Questionnaire 12; ESAS: Edmonton Symptom Assessment Scale.

Table 5 presents quality of evidence (GRADE) and psychometric property ratings for each tool.

**Table 5.** Psychometric rating and quality of evidence (GRADE) per tool.

	Tool (*Study ref*)	Content validity	Structural validity	Internal consistency	Measure invariance	Reliability	Measure. error	Criterion validity	Construct validity	Responsiveness	Score (mean)	COSMIN grade
Comfort	Hospice Comfort Questionnaire (4-point)^18^			VERY LOW ?				VERY LOW ?	VERY LOW -		−1 (−0.33)	B
	Hospice Comfort Questionnaire (6-point)^18^			VERY LOW ?					VERY LOW -		−1 (−0.33)	B
Quality of Life	FACT-G ^22,23^	VERY LOW +							MODERATE +		4 (2)	B
	EORTC QLQ C30 Version 1 ^28^								*HIGH* *-*		−4 (−4)	C
	EORTC QLQ C30 Version 2^25^	No GRADE +/-									0	B
	EORTC QLQ C30 Version 3^29,30^	No GRADE +/−		*MODERATE* *?*				*LOW* *-* *1		VERY LOW + *2	−1 (−0.25)	B
	EORTC QLQ C30 Version 3 mod.^31^	No GRADE +/−		*MODERATE* *?*					*VERY LOW* *-*	*VERY LOW* *+* *3 *-* *4	−1 (−0.25)	B
	McGill Quality of Life (7-point)^24,25^	No GRADE +/−		VERY LOW ?					MODERATE -		−3 (−1)	B
	McGill Quality of Life (11-point)^26,27^	No GRADE +/−	MODERATE ?	HIGH ?					HIGH -	VERY LOW -	−5 (−1)	C
Psych distress	GHQ-12^19–21^		LOW +		VERY LOW. -			HIGH +			5 (1.67)	B
Symptom Distress	Rotterdam Symptom Checklist^32^								VERY LOW -		−1 (−1)	B
	ESAS Likert scale (Best describes)^25,33^	No GRADE +/−									0	B
	ESAS Likert scale (24-h)^31^	No GRADE +/−		MODERATE ?						*VERY LOW* -*5, ?*6, +*7	0	B
	ESAS-revised^33^	No GRADE +/−						*LOW* *+* *8			2 (1)	B

FACT-G: Functional Assessment of Cancer Therapy – General; EORTC QLQ C30: European Organization for Research and Treatment of Cancer-Quality of Life-Core 30; GHQ-12: General Health Questionnaire 12; ESAS: Edmonton Symptom Assessment Scale. COSMIN Grade: A – recommended for use. B – potential for recommendation. C – recommendation against use. Roman indicates full tool evaluation. Italics indicates subscale evaluation.

* ^1^ All subscales rated insufficient, apart from Social which rated sufficient, with Low or Very-Low GRADE

* ^2^ Subscale responsiveness evidence also sufficient and Very-Low GRADE

* ^3^ Role, emotional, cognitive, fatigue, appetite, sleep disturbance and dyspnoea rated sufficient with Very-Low GRADE.

* ^4^ All other subscales and symptoms rated insufficient with Very-Low GRADE

* ^5^ Nausea, depression, pain, wellbeing and drowsiness rated as – at Very-Low GRADE.

* ^6^ Appetite, shortness of breath and fatigue rated ? at Very-Low GRADE.

* ^7^Anxiety rated as + at Very-Low GRADE.

* ^8^Wellbeing rated − with Very-Low GRADE.

### Psychometric properties of tools assessing comfort at the end-of-life

Two versions of the Hospice Comfort Questionnaire were evaluated in a single study.^18^ Psychometric assessments could not be combined as the tool was changed from a 6-point (version 1) to a 4-point scale (version 2). This was as authors were concerned that 6-response options were too many. Internal consistency and construct validity were assessed for both tools, and criterion validity assessed for version 2. Despite reporting promising Cronbach alpha values of 0.98 and 0.83, both tools were rated as indeterminate, as no evidence for structural validity was reported. Evidence of internal consistency was graded as Very-Low quality due to limited number of studies and small sample size (*n* = 48 and *n* = 38).

Hypothesis testing for construct validity was conducted and rated as insufficient for both versions as only two of six pre-defined hypotheses were met. Patient ratings appropriately correlated with caregiver ratings; however, tool scores did not adequately correlate with an alternative comfort measure at two time-points. GRADE of construct validity evidence was Very-Low as only one study with a sample of less than 100 was included. Lastly, criterion validity of version 2 was rated as indeterminate due to use of an inappropriate statistical test. This was deemed Very-Low quality evidence due to the single study, poor methodological quality and limited sample size (*n* = 86). Both versions of the Hospice Comfort Questionnaire received a global score of −1.

### Psychometric properties of General Health Questionnaire-12

The GHQ-12, a tool assessing psychological distress, was evaluated in three studies and data were combined.^19–21^ While the Rasch analysis provided insufficient evidence for structural validity, confirmative factor analysis was deemed sufficient.^19^ Measurement invariance was assessed by investigating differential item functioning. While all items functioned similarly between participants of different ages, a gender difference was present for two items (contrast = 1.82, *p* = 0.05) resulting in a rating of insufficient.^19^ This was graded as Very-Low quality as only one study of low methodological quality was included. Criterion validity was assessed in two studies.^20,21^ Psychometric evidence was sufficient as studies reported Receiver Operating Characteristic values of 0.81 and 0.76 which indicate strong positive correlations with the gold standard measure. GHQ-12 was administered via touchscreen monitors in two studies.^19,21^ The high completion rate and quick completion times suggest this tool and method of administration are acceptable for people with advanced disease. GHQ-12 received an overall score of 5, the highest across all tools.

### Psychometric properties of tools assessing Quality of Life

Tools assessing quality of life included FACT-G, two versions of the McGill Quality of Life Questionnaire and four versions of EORTC-QLQ C30. Development of the FACT-G involved people with advanced disease.^22^ Content validity was deemed to be sufficient overall; the only tool evaluated to receive this rating for content validity. This was a Very-Low GRADE however due to the single study being of inadequate quality and using a limited sample size (*n* = 45).^22^ Construct validity of FACT-G was evaluated through hypothesis testing.^23^ Pain scores appropriately correlated with Brief Pain Inventory and Memorial Symptom Assessment Scale thus meeting all seven hypotheses and resulting in a rating of sufficient. This was of Moderate quality as the single study had a large sample size (*n* = 238) and was of very good methodological quality. FACT-G received an overall score of 4, reporting no evidence of insufficient psychometric properties. Based on COSMIN guidance, the FACT-G can be provisionally recommended for use in this population, however internal consistency must be established before firm recommendations are made.

The McGill Quality of Life Questionnaire was assessed in four studies. The original version, using a 7-point rating scale, was evaluated in two studies.^24,25^ Promising Cronbach’s alpha values were reported in people living with advanced cancer (0.73) and AIDS (0.76).^24^ However as no evidence for structural validity was provided, an indeterminate rating was assigned for internal consistency. This was deemed Very-Low quality due to inadequate methodology quality. Hypothesis testing was used to evaluate construct validity by correlating the five subscales. As less than 75% of hypotheses (3/20) were met, psychometrics were rated as insufficient. This evidence was of Moderate methodological quality. Content validity was explored in one study,^25^ however due to an inadequate risk of bias, psychometric properties could only be rated as indeterminate. Content validity was rated as inconsistent overall as although the tool was comprehensive and relevant, the wording of some items and response options was deemed inappropriate. The McGill Quality of Life questionnaire using a 7-point rating scale received a global score of −3.

The updated 11-point rating scale version of McGill questionnaire was evaluated in two studies.^26,27^ Structural validity was indeterminate as, despite conducting explorative factor analysis, factor loadings were not reported. Responsiveness was evaluated by comparing standardised response means for intervention (0.14) and control groups (0.13), however these did not meet the threshold for ‘small’ differences so received a rating of insufficient. Content validity was rated as inconsistent as although comprehensiveness was deemed sufficient, it was considered that comprehensibility could be improved. Data were combined for internal consistency.^26,27^ Cronbach values greater than 0.7 were reported for the overall tool and subscales, however due to the lack of evidence of structural validity it was rated as indeterminate. Data on hypothesis testing for construct validity were combined.^26,27^ Forty correlations were conducted between the overall tool and other quality of life measures, symptom distress measures, depression measures and individual subscales of participant evaluated problem scores. As less than 75% of hypotheses were met (27/40), psychometric evidence for construct validity was rated insufficient. Due to multiple studies and low risk of bias, these data were deemed to be High quality. This tool is not recommended for use due to High quality evidence for psychometric insufficiency.

Four versions of EORTC-QLQ C30 were assessed (version 1, version 2 incorporating three alternative items, version 3 incorporating a 4-point response scale and a reworded item, and version 3 modified incorporating a recall period of 24-h instead of 1-week). Construct validity of version 1 was rated as insufficient as less than 75% of hypotheses relating to each subscale were met (ranging from 20% to 60% per subscale).^28^ The study was deemed High quality as it used a large sample size (*n* = 964) and had a risk of bias rating of very good. This tool is not recommended for use in advanced disease populations due to the high quality evidence for insufficient measurement properties. Content validity of version 2 was assessed in one study.^25^ The tool addressed the symptoms most frequently reported by people receiving palliative care, and concluded it was the most comprehensive of tools evaluated. However, based on criteria for good measurement properties, content validity received an overall rating of inconsistent as not all relevant concepts (such as vertigo, weight loss, cough and psychosocial issues) were included.

Content validity of version 3 was rated as inconsistent as it was deemed comprehensiveness could be improved.^29^ Internal consistency of social, emotional, role, global quality of life, fatigue and pain subscales was promising with Cronbach’s alpha values greater than 0.7.^30^ Low alpha values were reported for cognitive (0.19), physical (0.62) and nausea and vomiting (0.68) subscales indicating low internal consistency.^30^ However, as no evidence for structural validity was provided, an overall rating of indeterminate was given. This evidence is of Moderate quality due to very good methodological quality but a small sample size (*n* = 57). Hypothesis testing for construct validity resulted in a psychometric rating of sufficient for the social subscale (9/11 hypotheses met).^30^ All other functional scales and the fatigue and pain scales received insufficient ratings as, while some hypotheses were supported by data, less than 75% were met.^30^ Responsiveness was rated as sufficient as the two hypothesis were met.^29^ This was deemed to be Very-Low quality, due to a single study of doubtful quality and limited sample size (*n* = 65). Responsiveness of symptom items/subscales were also evaluated, and all received sufficient ratings of Very-Low quality.^29^ Luo et al. concluded that the tool would be suitable for use in clinics or trials conducted in Singapore due to the carefully selected wording.^30^ However, the study by Gough et al. highlighted that while the tool addressed some of the most common symptoms for people with advanced cancer, quantitative findings did not always align with participants’ qualitative reports.^29^

Results of modified version 3 could not be combined with original version 3 as the recall period differed. Content validity of modified version 3 received an inconsistent rating as relevance and comprehensibility were deemed sufficient, yet comprehensiveness was deemed to be lacking.^31^ Cronbach’s alpha values greater than 0.70 at baseline and follow up were reported for all subscales (excluding cognitive function) indicating good internal consistency.^31^ However internal consistency was rated indeterminate, as no data on structural validity were provided. Hypothesis testing for construct validity was conducted for subscales and symptom items. As less than 75% of hypotheses were met, the evidence was insufficient. Due to inadequate risk of bias rating, hypothesis testing evidence was graded Very-Low. Sufficient psychometric ratings were given for responsiveness data for role, emotional and cognitive subscales, and fatigue, appetite, sleep disturbance and dyspnoea symptom items. All other subscales and symptoms provided insufficient evidence. Responsiveness evidence received a GRADE of Very-Low due to limited sample size (*n* = 44) and inadequate risk of bias rating. Modified version 3, when used in combination with the pancreatic cancer specific module (QLQ-PAN26), was said to provide the broadest picture of change, however Easson et al. also noted that the combined tools provided redundant information and correlated poorly with participant reports.^31^ Global scores for EORTC QLQ C30 were; version 1 scored −4, version 2 scored 0, version 3 scored −1, and modified version 3 scored −1.

### Psychometric properties of tools assessing Symptom Distress

Tools assessing symptom distress included Rotterdam Symptom Checklist and three versions of ESAS; Likert scale, Likert modified and ESAS-revised. Construct validity of the Rotterdam Symptom Checklist was assessed through hypothesis testing where items of nausea, depression, anxiety, appetite and shortness of breath were correlated against corresponding items on ESAS.^32^ All five hypotheses were not met as kappa correlations were only at a moderate level, resulting in a rating of insufficient. This was Very-Low quality due to being a single study, low methodological quality with limited sample size (*n* = 80). The checklist received an overall score of −1.

Content validity of ESAS Likert scale was evaluated in two studies.^25,33^ While content relevance was promising, with 7/12 most frequent symptoms for people in palliative care addressed by ESAS, comprehensiveness was thought to be lacking resulting in an overall rating of inconsistent. In the Watanabe et al. study,^33^ participants described difficulty understanding ESAS terminology, such as distinguishing between ‘tiredness’ and ‘drowsiness’. The modified ESAS Likert version used a different recall period of ‘over the last 24 h compared to the original version, which doesn’t state a timeframe. Content validity was evaluated by Easson et al.^31^ resulting in an inconsistent rating. Cronbach’s alpha values were sufficient at baseline (0.80) and follow up (0.87), however internal consistency was rated as indeterminate as no structural validity data were provided. Responsiveness of individual subscales was evaluated by comparing mean change scores before and after an intervention. Subscales of depression, pain, wellbeing, drowsiness and nausea received psychometric ratings of insufficient as changes were non-significant. Appetite, shortness of breath and fatigue received indeterminate ratings as, while statistically significant effects were seen, it was unclear if these were clinically meaningful. The anxiety subscale received a sufficient rating however as the tool correlated with participant-reported change in symptoms (*r* = −0.46, *p* = 0.007). All subscale evidence received a GRADE of Very-Low due to inadequate risk of bias rating and risk of imprecision (*n* = 44).

ESAS-revised is an updated version of ESAS which; specifies recall period as ‘now’, includes definitions of each item, has a different item order, provides an example ‘other’ item and no line above the numbered scale. Content validity was assessed by Watanabe et al. who found comprehensibility to be sufficient.^33^ Findings were rated as indeterminate because it was deemed that comprehensiveness could be improved by the inclusion of social or functional scales. Individual subscales of ESAS-r were evaluated for criterion validity by conducting intraclass correlations between patient scores on the original ESAS and ESAS-r. Subscales of Depression, Appetite, Pain, Tiredness and Drowsiness all received a sufficient psychometric rating as correlations were between 0.74 and 0.83. The wellbeing subscale received an insufficient rating as the correlation reached only 0.65. Evidence GRADE was Low for each subscale due to doubtful risk of bias. While participants rated both versions of ESAS as easy to understand, ESASr was deemed significantly easier (*p* < 0.001) and preferred (*p* < 0.001). Watanabe et al. concluded that ESASr could replace original ESAS, however noted that further validity research was needed.^33^ The original and modified ESAS Likert received overall scores of 0 due to inconsistent psychometric properties. ESAS-r received a score of 2.

## Discussion

### Main findings

A two-stage systematic review was conducted to identify and evaluate which tool(s) may be best to use in trials of massage, reflexology and aromatherapy style therapies with people living with advanced disease. Despite a substantial number (*n* = 66) of trials and a wide range of multi-domain tools (*n* = 40), only seven of these were developed and/or validated in people living with advanced disease. Data synthesis was limited due to use of different versions of tools and study-specific adaptions. Study evidence was mostly of Very-Low to Low quality according to GRADE criteria, or in many cases, data were contradictory and could not be evaluated for trustworthiness.

GHQ-12 received the highest global score, followed closely by FACT-G. While GHQ-12 scored highest it had evidence of insufficient measurement invariance (albeit a Low level). Overall, no tools met COSMIN criteria for recommendation for use. As FACT-G received the only sufficient rating for content validity, it can be provisionally recommended for use in future trials of massage, reflexology and aromatherapy style complementary therapies in populations with advanced disease. Further research is needed to evaluate psychometric properties of the FACT-G and GHQ-12 in this population. If sufficient internal consistency is established, FACT-G could be recommended more firmly. The development and validation of a tool specifically for complementary therapy assessment in people living with advanced disease may be necessary as evidence suggests current alternatives are lacking.

### Strength and weaknesses

An extensive search of published literature was conducted to identify tools used in evaluation of massage, reflexology and aromatherapy in a broad range of clinical populations. This initial wide scope allowed for identification of tools beyond those used currently in palliative care.^5^ The rigorous COSMIN process^12^ allowed for fair and objective recommendations about the use of tools and highlighted their limitations.

There were some limitations to the review. Data synthesis was not possible in most cases due to use of different tool versions and study specific adaptions. ESAS and EORTC-QLQ C30 in particular were modified frequently to meet study needs. Watanabe et al., referring to ESAS, emphasised the need to develop a standardised tool and administration process for research to avoid these issues.^33^ We assessed each tool individually, and despite their variations, attempted to provide an overview of psychometric quality. Additionally, as only studies published in English were included (for pragmatic reasons) there is a risk other potentially relevant tools were missed.

Despite the broad search, few trials involving populations with advanced disease were identified. Some common palliative care specific outcome measure tools, such as EORTC-PAL and Palliative care Outcome Scale were not captured by our search as they have not been used to evaluate massage, reflexology or aromatherapy in the populations of interest.^34,35^ Other reviews have explored the psychometric properties of such tools for specified outcomes and in specified populations.^10,36,37^ However, a recent review found a mismatch between qualitative accounts of therapy effects and the domains covered by commonly used tools.^9^ This work suggests that tools currently used in palliative care research are not appropriate for the evaluation of massage, aromatherapy-massage or reflexology type interventions. By looking beyond palliative specific tools we hoped to identify alternative outcome measures which may be suitable for the assessment of complementary therapy effects.

### What this study adds

The psychometric properties of the seven identified tools which were developed or validated in populations with advanced disease, were mostly unclear or contradictory. Low scores arose due to the ‘worst score counts’ principle applied using the risk of bias assessment,^38^ where one mis-step in research or reporting can greatly impact resulting scores. All studies received an indeterminate rating for internal consistency despite reporting Cronbach values which met sufficiency criteria, due to lack of reported evidence on tool structural consistency. Rosenkoetter and Tate noted a similar reduction in ratings due to this principle when applying the COSMIN checklist to other tools.^39^ Poor quality ratings were also common, mostly due to small sample sizes. This is common within palliative care research as participants can be difficult to recruit due to; poor health, cognitive impairment or gatekeeping, and a greater rate of attrition due to deterioration and death.^7^ Evidence quality was also affected by the limited number of studies evaluating psychometric properties. Different versions of tools had to be independently evaluated. The volume of evidence per tool was reduced resulting in corresponding reductions on the GRADE scale. There is a need for research of greater quality (or better reporting) to improve COSMIN scores, and the consistent use of non-modified outcome tools, to establish validity and reliability.

At present there is no standardised tool for evaluation of complementary therapies in populations with advanced disease. Tools currently used in trials in palliative care, do not cover the domains identified from a qualitative evidence syntheses as important to people with advanced disease, which include; Wellbeing, Escapism, Longer term benefits, Benefits of the therapist and Overall experience.^6,9^ From the five domains, the FACT-G only captures aspects of Wellbeing and Escapism.^6^ Existing tools may therefore be limited in content as well as psychometric qualities. Despite the focus on only two domains, the FACT-G may be sensitive to therapy effects. The Functional Assessment of Cancer Therapy – Breast (a tool which contains the same domains as the FACT-G but with additional breast cancer questions) displayed trends towards significance in a previous RCT of reflexology with advanced cancer patients.^8^ As samples were smaller than required for statistical power, the trend towards improvement may suggest some sensitivity to complementary therapy effects. Although studies in the qualitative evidence synthesis had methodological limitations, further qualitative work to explore the mechanisms of impact would be helpful. It may also help to first understand which outcomes are the most important to assess, so that further research validating work can be targeted effectively. By developing a core outcome set for complementary therapies in palliative care, we can identify outcome which are meaningful to key stakeholders and relevant to complementary therapies. By consulting patients, complementary therapists and academic field experts we can identify a range of outcomes, then apply consensus methods to reach agreement on which outcomes are core. Incorporating the patient voice throughout development may help bridge the gap between patient experience and clinical trials. Consistent measurement of the outcome set in research may allow data to be better compared and combined in meta-analysis, and help us draw firmer conclusions on effectiveness.

Until a core set of outcomes is agreed, research should continue using tools which have been validated in the population of interest. While the FACT-G may not be the perfect tool for the evaluation of complementary therapies, it’s promising psychometric quality and focus on two of the five patient reported outcomes, may make it a suitable substitute until better alternatives become available. Concerns have been raised on the number of tools available in palliative care research. In a European survey of 311 professionals in palliative care, over 100 tools were identified as being used in research, though the majority of these had been cited less than 10 times.^40^ The identification of existing tools which may be improved through further validation and adaptions (such as to address additional domains) may avoid the development of another tool which lacks suitable validation. In future, the FACT-G could be adapted to capture the domain of ‘Overall experience’ (whether the therapy felt rewarding or was a positive experience) as no tool in the Armstrong et al. review addressed this.^9^

This review provided a thorough and rigorous assessment of multi-domain tools currently used in evaluation of massage, reflexology and aromatherapy complementary therapies in clinical populations. While further validation of existing tools is conducted, or until a more comprehensive and valid outcome measurement tool is developed, FACT-G is provisional recommended for use in future trials with people living with advanced disease.

## Supplemental Material

sj-pdf-1-pmj-10.1177_02692163221122955 – Supplemental material for How best to capture the impact of complementary therapies in palliative care: A systematic review to identify and assess the appropriateness and validity of multi-domain tools Supplemental material, sj-pdf-1-pmj-10.1177_02692163221122955 for How best to capture the impact of complementary therapies in palliative care: A systematic review to identify and assess the appropriateness and validity of multi-domain tools by Lucy Mitchinson, Christina Chu, Andrea Bruun, Ali-Rose Sisk, Megan Armstrong, Cecilia Vindrola-Padros, Nuriye Kupeli, Bridget Candy and Patrick Stone in Palliative Medicine
