# Supplementary material for: How best to capture the impact of complementary therapies in palliative care: A systematic review to identify and assess the appropriateness and validity of multi-domain tools
Source: Palliat Med. 2022 Sep 7;36(9):1320–35. doi: 10.1177/02692163221122955 (PMC9606018; doi:10.1177/02692163221122955)
Supplement: sj-pdf-1-pmj-10.1177_02692163221122955 – Supplemental material for How best to capture the impact of complementary therapies in palliative care: A systematic review to identify and assess the appropriateness and validity of multi-domain tools [file sj-pdf-1-pmj-10.1177_02692163221122955.pdf]

Supplementary Material: PROSPERO registration adjustments

The protocol was preregistered on PROSPERO (CRD42020161199). Only minor amendments to the protocol were made. Originally a third aim was included; to explore which outcome measurement tools (used in trials identified in search 1) detected a significant effect. This data was extracted but not reported in this paper. The protocol stated that trials identified in search 1 would be evaluated using the Cochrane Risk of Bias checklist, and only tools from moderate or high quality trials would be included in search 2. On reflection exclusion of tools based on this criteria was not deemed necessary as the methodological quality of the trial wouldn't affect the tool itself. Lastly, on reconsideration data was not presented by tool type (e.g. idiographic or nomothetic) or by therapy assessed. It was more practical to present data by tool domain for comparison.

Supplementary Material: Full search strategy for search 1

CINAHL	
Keywords	reflexology OR zone therap* OR reflex therap* OR foot reflex OR Massotherapy OR Massag* OR Soft tissue mobile?ation OR Soft tissue manipulation OR Swedish massage OR foot massag* OR feet massag* OR reflexotherapy OR aromatherapy OR Aroma OR Essential oil OR Plant oil OR Essence oil OR relaxation technique OR bodywork OR Craniosacral OR Rolfing OR shiatsu OR healing touch OR acupressure
Subject Headings	MH “Massage+” OR MH “Reflexology”<br>MH “Aromatherapy” OR MH “Aromatherapist” OR MH “Massage therapist” OR MH “Reflexologist”
Filters	English Language<br>Age group: All Adult<br>Publication type: Clinical trial, Randomised Control trial<br>Human
Date	No limit – 22/11/2019<br>Updated search: 09/06/2021

MEDLINE	
Keywords	reflexology OR zone therap* OR reflex therap* OR foot reflex OR Massotherapy OR Massag* OR Soft tissue mobile?ation OR Soft tissue manipulation OR Swedish massage OR foot massag* OR feet massag* OR reflexotherapy OR aromatherapy OR Aroma OR Essential oil OR Plant oil OR Essence oil OR relaxation technique OR bodywork OR Craniosacral OR Rolfing OR shiatsu OR healing touch OR acupressure
Subject Headings	Exp Massage/<br>Aromatherapy/<br>Reflexotherapy/
Filters	English Language<br>Humans<br>Age group: All Adult (19 plus years)<br>Publication type: Clinical trial, all OR evaluation studies OR multicentre study
Date	Inception 1946 – Nov 21 2019.<br>Updated search: 09/06/2021

EMBASE	
Keywords	reflexology OR zone therap* OR reflex therap* OR foot reflex OR Massotherapy OR Massag* OR Soft tissue mobile?ation OR Soft tissue manipulation OR Swedish massage OR foot massag* OR feet massag* OR reflexotherapy OR aromatherapy OR Aroma OR Essential oil OR Plant oil OR Essence oil OR relaxation technique OR

	bodywork OR Craniosacral OR Rolfing OR shiatsu OR healing touch OR acupressure
Subject Headings	Exp massage/<br>Reflexology/<br>Aromatherapy/
Filters	English Language<br>Human<br>Age group: Adult <18 to 64 years> & Aged <65+ years><br>Clinical Trials (All options): Clinical trial, Randomised Controlled trial or controlled clinical trials or multicentre study or phase 1 clinical trial or phase 2 clinical trial or phase 3 clinical trial or phase 4 clinical trial.
Date	Inception 1980 to 2019 Week 46.<br>Updated search: 09/06/2021

CENTRAL	
Keywords	reflexology OR "zone therap*" OR "reflex therap*" OR "foot reflex" OR Massotherapy OR Massag* OR "Soft tissue mobile?ation" OR "Soft tissue manipulation" OR "Swedish massage" OR "foot massag*" OR "feet massag*" OR reflexotherapy OR aromatherapy OR Aroma OR "Essential oil*" OR "Plant oil*" OR "Essence oil*" OR "relaxation technique" OR bodywork OR Craniosacral OR Rolfing OR shiatsu OR "healing touch" OR acupressure
Subject Headings	Massage explode all trees<br>Reflexotherapy explode all tress<br>Aromatherapy explode all trees
Filters	Trials
Date	No limit – 22/11/2019<br>Updated search: 09/06/2021

AMED	
Keywords	reflexology or zone therap* or reflex therap* or foot reflex or Massotherapy or Massag* or Soft tissue mobile?ation or Soft tissue manipulation or Swedish massage or foot massag* or feet massag* or reflexotherapy or aromatherapy or Aroma or Essential oil or Plant oil or Essence oil or relaxation technique or bodywork or Craniosacral or Rolfing or shiatsu or healing touch or acupressure
Subject Headings	Acupressure/<br>Massage/<br>Reflexology/<br>Exp Aroma Therapy/
Filters	English

Date	No limit – 04/12/2019<br>Updated search: 09/06/2021

Supplementary material: Full search strategy for search 2

Keywords<br>(Title and Sbtract)	palliat* or terminal* or endstage or hospice* or (end adj3 life) or (care adj3 dying) or ((advanced or late or last or end or final) adj3 (stage* or phase))<br>AND<br>(Short-form 36 Health Survey Scale) or SF-36 or (European Organization for Research and Treatment of Cancer Quality of Life Questionnaire) or (EORTC QLQ-C30) or EORTC or (EORTC QLQ-BR23) or (EORTC QLQ-CR29) or (EORTC QLQ CIPN20) or (Profile of Moods States) or POMS or (Functional Assessment of Cancer Therapy) or FACT or FACIT or FACT-B or FACT-G or FACT-BMT or (Hospice comfort questionnaire) or (Medical Outcomes Survey Short Form-8) or SF-8 or (Multidimensional Quality of Life Scale - Cancer) or MQOLS-CA or (Kidney Disease and Quality of Life Short-Form) or KDQOL-SF or MDASI or (Fibromyalgia Impact Questionnaire) or FIQ or (Pittsburgh Sleep Quality Index) or PSQI or (Life Satisfaction Questionnaire) or LSQ or (General Health Questionnaire) or GHQ-28 or (Hamburg Quality of Life Questionnaire in Multiple Sclerosis) or HAQUAMS or (Neuropathic Pain Impact on Quality-of-Life Questionnaire) or NePIQoL or (Quality of Life in Epilepsy) or QOLIE-31 or (Measure Yourself Concerns and Wellbeing) or MYCAW or (Patient Reported Outcomes Measurement Information System) or PROMIS or (Body-Mind-Spirit Well-being Inventory) or BMSWBI or (World Health Organisation Wellbeing Index) or WHO-5 or (Quality of Life Index) or QLI or(Rotterdam Symptom Checklist) or RSCL or (WHO Quality of Life-BREF) or WHOQOL-BREF or (Diabetes-Specific Quality of Life Scale) or DSQLS or WHOQOL-HIV or (Edmonton Symptom Assessment System Revised) or ESASr or (Tension and Profile of Mood Scale) or (Berlin Mood Questionnaire) or BSF or (General Health Self-Assessment Form) or QL0500 or (Quality of Life Scale) or QoLS OR (M.D. Anderson Symptom Inventory) or “MD anderson Symptom inventory” OR (World health Organisation Quality of Life HIV AIDS) or (Quality of Life Related to Dietary Change Questionnaire) or (QOL Related to Dietary Change) or (Perianesthesia Comfort Questionnaire) or (Osteoarthritis knee and hip quality of life) or OAKHQoL or (Asthma quality of life questionnaire) or (AQLQ) or (EuroQol-5D) or EQ-5D or (McGill Quality of Life Questionnaire) or MQOL<br><br>AND

	internal consistency/ or exp item total correlation/ or exp kuder richardson coefficient/ or exp split half correlation/ or exp test retest reliability/ or exp validity/ or exp concurrent validity/ or exp construct validity/ or exp content validity/ or exp criterion related validity/ or exp discriminant validity/ or exp face validity/ or exp multitrait multimethod/ or exp reproducibility/ or exp discriminant analysis/ or psychometr$.ti,ab. or clinimetr$.ti,ab. or clinometr$.ti,ab. or reproducib$.ti,ab. or reliab$.ti,ab. or unreliab$.ti,ab. or valid$.ti,ab. or coefficient.ti,ab. or homogeneity.ti,ab. or homogeneous.ti,ab. or "internal consistency".ti,ab. or (cronbach$ and (alpha or alphas)).ti,ab. or (item and (correlation$ or selection$ or reduction$)).ti,ab. or agreement.ti,ab. or precision.ti,ab. or imprecision.ti,ab. or "precise values".ti,ab. or test retest.ti,ab. or (test and retest).ti,ab. or (reliab$ and (test or retest)).ti,ab. or stability.ti,ab. or interindividual.ti,ab. or inter-individual.ti,ab. or intraindividual.ti,ab. or intra-individual.ti,ab. or interparticipant.ti,ab. or inter-participant.ti,ab. or intraparticipant.ti,ab. or intra-participant.ti,ab. or kappa.ti,ab. or kappas.ti,ab. or kappas.ti,ab. or repeatab$.ti,ab. or ((replicab$ or repeated) and (measure or measures or findings or result or results or test or tests)).ti,ab. or generaliza$.ti,ab. or generalisa$.ti,ab. or concordance.ti,ab. or (intraclass and correlation$).ti,ab. or discriminative.ti,ab. or "known group".ti,ab. or factor analysis.ti,ab. or factor analyses.ti,ab. or dimension$.ti,ab. or subscale$.ti,ab. or (multitrait and scaling and (analysis or analyses)).ti,ab. or item discriminant.ti,ab. or interscale correlation$.ti,ab. or error.ti,ab. or errors.ti,ab. or "individual variability".ti,ab. or (variability and (analysis or values)).ti,ab. or (uncertainty and (measurement or measuring)).ti,ab. or "standard error of measurement".ti,ab. or sensitiv$.ti,ab. or responsive$.ti,ab. or ((minimal or minimally or clinical or clinically) and (important or significant or detectable) and (change or difference)).ti,ab. or (small* and (real or detectable) and (change or difference)).ti,ab. or meaningful change.ti,ab. or "ceiling effect".ti,ab. or "floor effect".ti,ab. or "item response model".ti,ab. or IRT.ti,ab. or rasch.ti,ab. or "differential item functioning".ti,ab. or DIF.ti,ab. or "item bank".ti,ab.<br>NOT<br>(addresses or biography or case reports or comment or directory or editorial or festschrift or interview or lectures or legal cases or legislation or letter or news or newspaper article or patient education handout or popular works or congresses or consensus development conference or consensus development conference, nih or practice guideline or Poetry or editorial or encyclopedia entry or interview or letter or Obituary)
Subject Headings	exp palliative care/

	terminally ill patients/
Filters	English Language<br>Humans
Date	Inception 1806 - 03/06/2020<br>Updated: 2020 – 24/08/21

Supplementary material: ‘Good Measurement Properties’ criteria used to assess psychometric properties[9]

Psychometric Property	Rating	Quality Criteria
Structural validity	+	Classic test theory:<br>Confirmatory factor analysis: Comparative fit index or Tucker-Lewis index or comparable measure >0.95 OR Root Mean Square Error of Approximation (RMSEA) <0.06 OR Standardized Root Mean Residuals <0.08<br><br>Item response theory/Rasch:<br>No violation of uni-dimensionality: Comparative fit index or Tucker-Lewis index or comparable measure >0.95 OR Root Mean Square Error of Approximation <0.06 OR Standardized Root Mean Residuals <0.08<br>AND<br>No violation of local independence: residual correlations among the items after controlling for the dominant factor <0.20 OR Yen’s Q3’s <0.37<br>AND<br>No violation of monotonicity: adequate looking graphs OR item scalability >0.30<br>AND<br>Adequate model fit:<br>Item response theory: $\mathcal{X}^2$ >0.01<br>Rasch: infit and outfit mean squares ≥ 0.5 and ≥ 1.5 OR Z-standardized values > -2 and <2
	?	Classic test theory: Not all information for ‘+’ reported<br>Item response theory/Rasch: Model fit not reported
	-	Criteria for ‘+’ not met
Internal consistency	+	At least low level evidence for sufficient structural validity AND Cronbach’s alpha(s) ≥ 0.70 for each unidimensional scale or subscale
	?	Criteria for “at least low level evidence for sufficient structural validity” not met
	-	At least low level evidence for sufficient structural validity AND Cronbach’s alpha(s) < 0.70 for each unidimensional scale or subscale
Reliability	+	Intraclass correlation coefficient or weighted Kappa ≥ 0.70
	?	Intraclass correlation coefficient or weighted Kappa not reported
	-	Intraclass correlation coefficient or weighted Kappa < 0.70
Measurement Error	+	Smallest detectable change or Limits of agreement < minimal important change
	?	Minimal important change not defined

	-	Smallest detectable change or Limits of agreement > minimal important change
Hypothesis testing for construct validity	+	The results in accordance with the hypothesis set by the review team.
	?	No hypothesis defined by the review team
	-	The results are not in accordance with the hypothesis
Measurement invariance	+	No important differences found between group factors (such as age, gender, language) in multiple group factor analysis OR no important differential item functioning (DIF) for group factors (McFadden's R^2 <0.02)
	?	No multiple group factor analysis OR differential item functioning was found
	-	Important differences between group factors OR differential item functioning was found
Criterion validity	+	Correlations with gold standard ≥ 0.70 OR Area under curve ≥ 0.70
	?	Not all information for '+' reported
	-	Correlation with gold standard < 0.70 or Area under curve < 0.07
Responsiveness	+	The result is in accordance with the hypothesis set by the review team OR Area under curve ≥ 0.70
	?	No hypothesis defined by the review team
	-	The result is not in accordance with the hypothesis OR Area under curve < 0.70
Content Validity	+	≥85% of the items of the PROM (or subscale) fulfil the criterion for Relevance, Comprehensiveness or Comprehensibility (criteria provided in Appendix 3).
	?	Not enough information available OR quality of part of the study is inadequate
	-	<85% of the items of the PROM (or subscale) does fulfil the criteria (criteria provided in Appendix 3).

Supplementary material: 10 criteria for good content validity

**Relevance**
1. Are the included items relevant for the construct of interest?
2. Are the included items relevant for the target population of interest?
3. Are the included items relevant for the context of use of interest?
4. Are the response options appropriate?
5. Is the recall period appropriate?
**Comprehensiveness**
6. Are all key concepts included?
**Comprehensibility**
7. Are the PROM instructions understood by the population of interest as intended?
8. Are the PROM items and response options understood by the population of interest as intended?
9. Are the PROM items appropriately worded?
10. Do the response options match the question?

Supplementary material: Table of included papers search 1

	Reference	Country	Population	Sample Size	Therapy	Outcome measure	Setting	Aim
1	Ahles et al., 1999	USA	Bone marrow transplant	32	Massage	State Trait Anxiety Inventory (STAI).<br><br>Beck Depression Inventory (BDI).<br><br>**Brief Profile of Mood States (POM-SF).**	Medical Centre	Examine the feasibility of conducting a randomized, clinical trial of massage therapy in a bone marrow transplant setting and to test the efficacy of massage therapy in reducing symptoms of physical and psychological distress.
2	Arab et al., 2015	Iran	Haemodialysis	108	Acupressure	**Pittsburgh Sleep Quality Index (PSQI).**<br><br>Sleep Chart.<br><br>**Short Form 13-item health survey questionnaire (SF-13).**	Hospital	Investigate the effect of acupressure on the sleep and life quality in patients undergoing haemodialysis by means of a controlled trial.
3	Berggreen et al., 2012	Denmark	Females with chronic tension-type headache	39	Massage	Pain Visual Analogue Scale.<br><br>McGill Pain Questionnaire.<br><br>**Short-form 36-item health survey questionnaire (SF-36).**	*unclear*	Evaluate the efficacy of myofascial trigger points massage in the muscles of the head, neck and shoulders regarding pain in the

								treatment of patients with chronic headaches.<br><br>Observe changes in medicine consumption and quality of life.
4	Bergmann et al., 2014	Denmark	Ischemic heart disease	181	Acupressure	**World Health Organisation-Five Well-Being Index (WHO-5).**<br><br>**Short-form 36-item health survey questionnaire (SF-36).**<br><br>Pressure Pain Sensitivity.<br><br>Major Depression Inventory.<br><br>Clinical Stress Symptoms.	Hospital	Test the hypothesis: The combination of daily self-measurements of Pressure Pain Sensitivity aiming at increased empowerment followed by acupressure aiming to restore diffuse noxious inhibitory control system, together would resolve in a reduction of the following elements of chronic stress: Depressive symptoms, general well-being and physically and mentally quality of life.

5	Bertolaccini Martínez et al., 2018	Brazil	Type 2 diabetes	62	Reflexology	Stanford Health Assessment Questionnaire Disability Index.<br><br>**Short-form 36-item health survey questionnaire (SF-36).**	Diabetes Education Center	Compare the effects of feet reflexology in quality of life and functional capacity of diabetic patients.
6	Billhult, A., et al. 2008	Sweden	Breast cancer	22	Massage	Phenotypic characterization of peripheral blood NK cells, Peripheral blood mononuclear cell (PBMC) isolation, T helper cells and cytotoxic T cells.<br><br>Neuroendocrine function, endocrine function, NK cell cytotoxicity.<br><br>Hospital Anxiety and depression scale (HADS).<br><br>State-Trait Anxiety Inventory (STAI).<br><br>**Life satisfaction questionnaire**.	Hospital	Study the effect of repeated effleurage massage on cellular immunity in patients with breast cancer.<br><br>Evaluate the effect of massage on cortisol, oxytocin, anxiety, depression and quality of life in patients with breast cancer.
7	Birk et al., 2000	USA	HIV	42	Massage	**General Health Self-Assessment form.**<br><br>Lymphocyte counts (CD4+, CD8+ & NK cells).	Academic medical centre, Red cross and other agencies for people with HIV/AIDs	Determine the effects of massage therapy alone and massage combined with exercise or stress management on enumerative immune measures and on six dimensions of quality of life in comparison

								to controls receiving standard treatment.
8	Blackburn et al., 2017	USA	Acute leukaemia patients suffering insomnia	50	Aromatherapy	**Pittsburgh Sleep Quality Index (PSQI).**<br><br>**Edmonton Symptom Assessment System-revised (ESAS-r).**<br><br>Final evaluation of aromatherapy form.	Acute leukemia unit of a hospital and Research Institute of a Medical Center	Determine if aromatherapy by diffusion of essential oils improves insomnia in hospitalized patients newly diagnosed with acute leukemia.<br><br>Determine (a) whether aromatherapy improves other common symptoms in this patient population and (b) whether patients perceive aromatherapy to be a positive experience for symptom management.
9	Bodhise et al., 2004	USA	Sickle cell disease	5	Massage	Numeric Pain Index Scales.<br><br>**Tension and Profile of Mood Scale (TPMS).**<br><br>Activities of Daily Living Assessment.	*unclear*	Evaluate whether deep tissue/deep pressure neuromuscular massage would reduce pain intensity, tension, opioid consumption and increase relaxation

								and activities of daily living.
10	Brygge et al., 2001	Denmark	Bronchial asthma	40	Reflexology	Diary cards (self-measured peak flows, symptom score, use of b2-agonist).<br><br>Lung function (forced expiratory volume 1/second and forced vital capacity).<br><br>Bronchial sensitivity (PC20 histamine).<br><br>**Short-form 36-item health survey questionnaire (SF-36).**	Allergy Unit	Study reflexology's effect on bronchial asthma assessed by clinical symptoms, medicine intake, objective lung function parameters, bronchial sensitivity and quality-of-life questionnaires.
11	Çevik & Tas¸cı, 2020	Turkey	Haemodialysis	50	Acupressure	Patient information form.<br><br>Pain VAS.<br><br>**Kidney Disease Quality of Life (KDQoL-36).**	Hospital	To examine the effect of the application of acupressure on upper extremity pain and Quality of Life in patients undergoing Haemodialysis treatment.
12	Cook et al., 2004	USA	Gynaecological or breast cancer receiving radiation treatment	62	Healing touch	Demographic information (standard sociodemographic interview).<br><br>Medical information.<br><br>Attitudes about Healing Touch.<br><br>Beliefs about group assignment.	Radiology oncology department	Test the primary hypothesis that women who receive healing touch during their radiation treatment for gynaecological or breast cancer will report better health-related quality of life

						**Short-form 36-item health survey questionnaire (SF-36).**		than their counter parts who receive mock treatment.
13	Dalala et al., 2013	India	Intractable epilepsy	77	Reflexology	**Quality of Life in Epilepsy Inventory (QOLIE-31).**	Neurology Outpatient Department of a tertiary care medical institute	Critically examine the efficacy of hand and foot reflexology therapy in treating epilepsy patients who fulfil the criteria of intractability.
14	Dikmen et al., 2019	Turkey	Gynaecologic cancer receiving chemotherapy	80	Reflexology	Brief Pain Inventory (BPI).<br><br>Brief Fatigue Inventory (BFI).<br><br>**Multidimensional Quality of Life Scale- Cancer.**<br><br>General data collection form.	Hospital	Determine the effects of reflexology and progressive muscle relaxation exercises on gynaecologic cancer patients' pain, fatigue, and quality of life during chemotherapy and their effects independent of analgesics and pain medications on gynaecologic cancer patients.
15	Dyer et al., 2013	UK	Cancer	115	Reflexology	**Measure Yourself Concerns and Wellbeing (MYCAW).**<br><br>VAS relaxation score.	Specialist cancer centre	Ascertain whether reflexology is as effective as aromatherapy massage for self-selected concerns.

								Assess the difference between groups in MYCaW second concerns, overall wellbeing scores and levels of relaxation. Collect patients' comments on the intervention they received.
16	Field et al., 2004	USA	Carpal tunnel syndrome	16	Massage	Perceived Grip Strength Scale.<br><br>VITAS pain assessment scale.<br><br>State anxiety inventory (STAI).<br><br>**The Profile of Mood States (POMS).**	University	Examine massage therapy effects on pain, median nerve conduction, and accompanying anxiety and depression related to Carpal Tunnel Syndrome.
17	Gok Mentin et al., 2017	Turkey	Painful diabetic neuropathy	46	Aromatherapy -massage	Patient questionnaire.<br><br>Douleur Neuropathique en 4 Questions (DN4).<br><br>Pain visual analogue scale.<br><br>**Neuropathic Pain Impact on Quality of Life (NePIQoL).**	University hospital endocrine outpatient clinic	Examine the effects of aromatherapy massage on neuropathic pain severity and quality of life in patients suffering from painful diabetic neuropathy.
18	Habibzadeh et al., 2019	Iran	Haemodialysis	120	Massage	Demographic questionnaire.<br><br>Fatigue Severity Scale.	Educational and medical centers located in two hospitals	Explore the impact of foot massage with chamomile oil and almond oil on the severity of fatigue

						**Kidney Disease Quality of Life-Short Form (KDQOL-SF)**		and quality of life of Haemodialysis patients.
19	Hasanpour-Dehkordi, Kabiri & Dris, 2021	Iran	Knee osteoarthritis	93	Massage and Aromatherapy	Demographic questionnaire<br><br>**Knee Injury and Osteoarthritis Outcome Score (KOOS)**	Hospital	Compare the effects of massage therapy and aromatherapy on knee pain, morning stiffness, daily routine function, and quality of life in patients with knee osteoarthritis.
20	hmwe et al., 2015	Malaysia	Haemodialysis	108	Acupressure	Depression Anxiety Stress Scale-21 (DASS-21).<br><br>**General Health Questionnaire-28 (GHQ-28).**	Haemodialysis centres	Evaluate the effects of acupressure on depression, anxiety, stress, and general psychological distress in a cohort of patients with haemodialysis in Malaysia.
21	Izgua et al., 2019	Turkey	Breast cancer (chemotherapy induced peripheral neuropathy)	40	Massage	Demographics form.<br><br>Self-Leeds assessment of neuropathic symptoms and signs (S-LANSS).<br><br>**European Organization for Research and Treatment of Cancer Quality of Life Questionnaire - Chemotherapy-**	Chemotherapy outpatient clinic of Health Sciences University Oncology Training and Research Hospital	Examine the effect of classical massage in breast cancer patients receiving adjuvant paclitaxel using nerve conduction studies combined with patient-reported

						**Induced Peripheral Neuropathy module (EORTCQLQ-CIPN20).**<br><br>Nerve conduction studies.		outcomes, in order to assess Chemotherapy-Induced Peripheral Neuropathy.
22	Ji-hua, X., et al. (2017)	China	Chronic migraine	98	Acupressure	Numeric pain rating scale, frequency of migraine attacks, frequency of analgesics use, and adverse events.<br><br>**Short-form 36-item health survey questionnaire (SF-36).**	Hospital	Evaluate the efficacy and safety of using acupressure as an adjunctive therapy to sodium valproate combined with acupressure on the prevention of chronic migraine with aura.
23	Jung & Chang, 2020	South Korea	Haemodialysis	60	Acupressure	Demographic questionnaire<br><br>Xerostomia VAS<br><br>Salivary flow rate<br><br>Interdialytic weight gain<br><br>Constipation Assessment Scale<br><br>**Quality of Life Related to Dietary Change Questionnaire**	Haemodialysis centres	Examine the effects of Acupressure on physical and mental health in patients on haemodialysis.
24	Kapıkıran & Özkan, 2021	Turkey	Liver transplantation	120	Reflexology	Patient Identity Form<br><br>Pain VAS	Organ transplantatio n clinic	Determine the effect of foot reflexology on pain, comfort and β-Endorphin levels of patients who

						Beta-Endorphin Level Entry Form<br><br>**Perianesthesia Comfort Questionnaire (PCQ)**		received liver transplantation.
25	Keskin & Taşci, 2021	Turkey	Haemodialysis	60	Acupressure	Pain VAS<br><br>Fatigue VAS<br><br>Severity of thirst VAS<br><br>**Quality of Life Scale**	Dialysis units of private hospitals	Determine the effect of acupressure applied to patients receiving Haemodialysis treatment on their severity of thirst and quality of life
26	Kolcaba et al., 2004	USA	Palliative care	31	Massage	**Hospice comfort questionnaire.**<br><br>Symptom distress scale.	Hospice agencies	Determine empirically if there are beneficial effects associated with hand massage done twice per week for 3 weeks on patients near end of life.
27	Kutner et al., 2008	USA	Advanced cancer	380	Massage	Memorial Pain Assessment Card.<br><br>Brief Pain Inventory.<br><br>60-second heart and respiratory rates.<br><br>**McGill Quality of Life Questionnaire.**	Hospices and university cancer center	Evaluate the efficacy of massage compared with an exposure controlling for time, attention, and touch.

						Memorial Symptom Assessment Scale.<br><br>Name, dose, and frequency of symptom management medications.<br><br>Adverse events.		
28	Lari et al., 2020	Iran	Diabetes mellitus type II and insomnia	52	Aromatherapy	Pittsburgh Insomnia Rating Scale-20 (PIRS-20),<br><br>**WHO Quality of Life-BREF (WHOQOLBREF)**<br><br>Beck Depression Inventory (BDI)<br><br>Calorie intake by 3 days food record questionnaire<br><br>International Physical Activity Questionnaires (IPAQ)<br><br>Enzymatic (glucose oxidase) colorimetric method<br><br>Body Mass Index (BMI),<br><br>Waist and hip circumferences.	Diabetes clinic	Evaluated its efficacy and safety on insomnia and the associated depression and decreased quality of life in patients with diabetes mellitus.
29	Listing et al., 2010	Germany	Breast cancer	34	Massage	Perceived Stress Questionnaire.<br><br>**Berlin Mood Questionnaire (BSF).**	Breast Cancer Center of a University Hospital	Evaluate the short and longer term effects of a standardized

						Endocrine measures.		classical massage on stress perception and mood disturbances as well as on serotonin and cortisol serum levels as physiological indicators.
30	Listing Reihauer et al., 2009	Germany	Breast cancer	86	Massage	**Short-form 8-item health survey questionnaire (SF-8).**<br><br>**European Organization for Research and Treatment of Cancer Quality of Life Questionnaire (EORTC QLQ-C30).**<br><br>Giessen Subjective Complaints List (GBB).<br><br>**Berlin Mood Questionnaire (BSF).**	Breast Cancer Center of a University Hospital	Evaluate whether massage therapy improves quality of life, including physical discomfort, fatigue, and mood disturbances.
31	Lu et al., 2016	USA	Hematopoietic stem cell transplant	46	Healing touch and Relaxation therapy	Date of admission, transplantation, engraftment, discharge and reasons for readmission to hospital during the 100 days after transplant.<br><br>Psychosocial assessment.<br><br>**Profile of mood states short form (POMS-SF)**	Adult Blood and Bone Marrow Transplant Unit of the University of Iowa Hospitals and Clinic	Identify the feasibility of utilizing Healing touch and Reflexology therapy with patients who had received stem cell transplant and to determine preliminary efficacy of these 2 interventions

						**The Functional Assessment of Cancer Therapy – Bone Marrow Transplant (FACT-BMT).**<br><br>Center for Epidemiologic Studies-Depression (CES-D).		by comparing clinical outcomes to a historical group of patients who had received usual care for stem cell transplant during the same year.
32	Lua et al 2015	Malaysia	Breast cancer	60	Aromatherapy	Severity of nausea and incidence of vomiting visual analogue scale.<br><br>**European Organization for Research and Treatment of Cancer Quality of Life Questionnaire (EORTC QLQ-C30).**	Oncology clinics	Determine the impact of aromatherapy using ginger essential oil in alleviating chemotherapy induced nausea and vomiting in breast cancer patients.<br><br>Assess patient’s health-related quality of life profile following aromatherapy treatments.
33	Lutgendorf et al., 2010	USA	Cervical cancer	60	Healing touch and Relaxation therapy	The Center for Epidemiological Studies Depression Scale (CES-D).<br><br>**Profile of Mood States-Short Form (POMS-SF).**<br><br>**The Functional Assessment of Cancer Therapy (FACT).**	Gynaecologic Oncology service at University Hospitals and Clinics	Examine effects of Healing Touch on NK cell activity, mood, and specific clinical and quality of life outcomes among women with locally advanced cervical cancer receiving a standard 6-week

						Modified Credibility of Therapy scale.<br><br>Blood pressure, clinical information and NK cell activity.		course of chemoradiation.
34	Lyu et al., 2019	China	Older adults with type 2 diabetes mellitus	66	Massage	Fasting blood glucose, 2-hour postprandial blood glucose and glycosylated haemoglobin.<br><br>**Diabetes-Specific Quality of Life Scale.**	Department of Endocrinology of a hospital	Explore self-acupoint massage for older adults with type 2 diabetes mellitus and examine its effect on blood glucose level and quality of life.
35	Macnamara et al., 2003	USA	Diagnostic cardiac catherization	46	Massage	Heart rate, heart rate variability, blood pressure, respiration, peripheral skin temperature and pain.<br><br>**Brief Profile of Mood States (POM-SF).**	Academic medical centre	Determine the effects of a 20-minute back massage on the physiological and psychological human response to cardiac patients admitted for a diagnostic cardiac catheterization.
36	Mei et al 2017	China	Coronary heart disease symptoms; awaiting coronary angiography	185	Massage	Hamilton Anxiety Rating Scale.<br><br>Blood pressure, heart rate, adverse events.<br><br>**Short-form 36-item health survey questionnaire (SF-36).**	Hospital	Evaluate the effectiveness and safety of Chinese hand massage care on anxiety among patients awaiting coronary angiography.
37	Mohammad pourhodki et al., 2021	Iran	Haemodialysis	105	Aromatherapy massage	Sociodemographic data questionnaire,<br><br>Fatigue Severity Scale (FSS),	Haemodialysis ward	Evaluate the effects of aromatherapy massage with

						**Pittsburgh Sleep Quality Index (PSQI),**<br><br>**36-Item Short Form Health Survey questionnaire (SF-36)**		Lavender and Citrus Aurantium essential oil on the quality of life in haemodialysis patients.
38	Molassiotisa et al., 2019	China	Depression	84	Acupressure	Geriatric Depression Scale.<br><br>**Pittsburgh Sleep Quality Index (PSQI).**<br><br>**The General Health Questionnaire (GHQ).**<br><br>**Body-Mind-Spirit Well-being Inventory.**<br><br>**World Health Organisation Quality of Life 28 items (WHOQOL-BREF).**	Care in the community	Assess the clinical effectiveness of acupressure in the management of depressive symptoms in elderly people compared to patients receiving sham acupressure or standard care alone.
39	Nadal-Nicolás et al., 2020	Spain	Women with Fibromyalgia	24	Manual therapy	Fatigue severity scale (FSS)<br><br>Pain VAS<br><br>**Pittsburgh sleep quality index (PSQI)**<br><br>**Profile of mood states (POMS-29**	Local rheumatology practices	Assess the effectiveness of a manual therapy technique performed with moderate digital pressure in Fibromyalgia patients on the variables of fatigue, pain, sleep, anxiety, and mood.

40	Nasiri et al., 2011	Iran	Haemodialysis (diagnosis of renal failure)	62	Acupressure	**Pittsburgh Sleep Quality Index (PSQI).**<br><br>Sleep log.	Hospital	Revaluate the effectiveness of acupressure on quality of sleep in haemodialysis patients.
41	Odebiyi et al., 2014	Nigeria	Breast cancer	27	Massage	**European Organization for Research and Treatment of Cancer ' s Quality of Life questionnaire (EORTC QLQ-C30) and Energy/Fatigue Scale.**	Outpatient unit of the Radio-therapy and Oncology Department of a University Teaching Hospital	Determine the effect of combined aerobic exercise and oedema massage on fatigue level and quality of life in female patients with breast cancer.
42	Ovayolu et al., 2014	Turkey	Breast cancer receiving chemotherapy	280	Aromatherapy and classic massage	**Rotterdam symptom checklist.**<br><br>**Quality of life scale.**	Chemotherapy unit of a private branch hospital	Assess the effect of aromatherapy and classic massage administered in various ways to breast cancer patients on their symptoms and quality of life.
43	Ozdelikara & Tan 2017	Turkey	Breast cancer	60	Reflexology	Patient identification forms.<br><br>**Quality of Life Subscale - European Organization for Research and Treatment of Cancer Quality of Life Questionnaires (EORTC QLQ) C30.**	Ambulatory chemotherapy unit of a University Faculty of Medicine Hospital	Identify the effect of reflexology on the quality of life in patients with breast cancer.

44	Pehlivan & Karadakovan, 2019	Turkey	Elderly with knee osteoarthritis	90	Aromatherapy massage	Patient information form<br><br>Western Ontario and McMaster Universities Osteoarthritis Index (WOMAC) Knee Osteoarthritis Evaluation Scale<br><br>**Osteoarthritis knee and hip quality of life (OAKHQoL)**	Nursing homes	Determine the effects of aromatherapy (black seed, ginger and rosemary oils) and massage on pain, functional state, and quality of life of elderly individuals with knee osteoarthritis.
45	Qi et al., 2018	China	Ankle pain accompanied by functional disorders	48	Massotherapy	Comparison of symptoms and physical signs (pain, tenderness, swelling, dysfunction and other symptoms and physical signs including local pain, local tenderness, swelling and dysfunction).<br><br>Pain severity assessment Visual Analogue Scale.<br><br>Kellgren and Lawrence classification under X-ray.<br><br>Mazur's ankle function score.<br><br>**European Organization for Research and Treatment of Cancer Quality of Life Questionnaires (EORTC QLQ) C30.**	University of Chinese Medicine Third Affiliated Hospital	Investigate the effect of ankle joint injection combined with the massotherapy of traditional Chinese medicine on functional recovery and quality of life of ankle joint osteoarthrosis patients

46	Razak et al., 2019	Malaysia	Chronic brachial neuralgia	40	Acupressure	The Brief Pain Inventory (BPI)<br><br>**Short Form 36 Health Survey (SF-36v2)**	University medical Centre	Assess and compare the efficacy of acupressure and hypnotherapy in terms of reducing pain intensity and improving the quality of life in patients presenting with Chronic Brachial Neuralgia following traumatic brachial plexus injury
47	Reychler et al., 2017	Belgium	HIV	29	Massage	Hospital Anxiety and Depression Scale (HADS).<br><br>Nijmegen questionnaire.<br><br>**World Health Organisation Quality of Life – HIV (WHOQOL-HIV)**	Out-patient infectious disease clinics	Compare in a randomized controlled study the effect of a 4 week massage therapy on anxiety, depression, hyperventilation and quality of life in HIV infected patients.
48	Rodrigues et al., 2018	Brazil	Fibromyalgia	24	Massage	**Fibromyalgia Impact Questionnaire.**<br><br>Perceived Stress Questionnaire.<br><br>McGill Pain Questionnaire (MPQ-Br).	Physio-therapy clinic of a University Center	Verify if a massage therapy program during three months can influence cortisol concentrations, perceived stress index, intensity of pain and quality of life of patients with Fibromyalgia

						Saliva to evaluate cortisol concentration before and after the end of each month.		syndrome before the treatment and after the end of each month.
49	Sajadi et al., 2020	Iran	Multiple sclerosis	63	Reflexology	Demographic questionnaire,<br><br>Constipation Assessment Scale<br><br>**Short Form 36 Quality of Life (SF-36)**	Multiple sclerosis society affiliated with a university	Investigate the effect of foot reflexology on constipation and quality of life in patients with Multiple sclerosis
50	Schroeder et al., 2014	Canada	Multiple Sclerosis	24	Massage	Patient comparative questionnaire and massage therapist questionnaire.<br><br>Six-Minute Walk Test.<br><br>**Hamburg Quality of Life Questionnaire in Multiple Sclerosis (HAQUAMS).**<br><br>Expanded Disability Status Scale.<br><br>Health assessment.	Hospital	Test the hypothesis that massage therapy will improve the leg function and overall quality of life of MS patients.
51	Shen et al., 2017	Australia	End-Stage Kidney Disease on Haemodialysis	42	Acupressure	Sleep diary.<br><br>**Short-form 8-item health survey questionnaire (SF-8).**<br><br>**Pitsburg Sleep Quality Index (PSQI).**	Hospital dialysis units	Investigate the effect of acupressure on the sleep quality of prevalent haemodialysis patients and establish the feasibility and safety of acupressure treatment in a

						Treatment acceptability questionnaire.		Western healthcare setting.
52	Sikorskii et al., 2020	USA	Breast cancer	256	Reflexology	Bayliss tool.<br><br>Center for Epidemiologic Studies-Depression.<br><br>**The M.D. Anderson Symptom Inventory.**	Community-based oncology clinics and cancer centres	Determine the effects of a 4-week, home-based reflexology intervention delivered by a friend/family caregiver compared to attention control on responses for multiple.<br><br>Explore which individual, disease, and treatment characteristics were associated with symptom responses
53	Soden et al., 2004	UK	Palliative care	42	Aromatherapy -massage	VAS of pain intensity.<br><br>Modified Tursky Pain Descriptors Scale.<br><br>Verran and Snyder-Halpern sleep scale.<br><br>Hospital Anxiety and Depression scale.<br><br>**Rotterdam Symptom Checklist**.	Specialist palliative care units	Evaluate the effects of a course of massage with and without an essential oil, on pain scores.<br><br>Test the hypotheses that these therapies<br>1. improve sleep quality<br>2. reduce anxiety and depression

								3. improve overall quality of life
54	Tabiee et a.., 2017	Iran	Haemodialysis	40	Massage	A demographic questionnaire (age, gender, marital status, educational status, and place of residence)<br><br>**Short form of hospice comfort questionnaire.**	Haemodialysis unit of a hospital	Evaluate the effects of comfort-based interventions (back massage and patient and family education) on the level of comfort among haemodialysis patients.
55	Tamaki et al., 2017	Japan	Breast cancer during perioperative periods	110	Aromatherapy	**European Organization for Research and Treatment of Cancer Quality of Life Questionnaires (EORTC QLQ) C30 version 3.**<br><br>Rates of the using hypnotics.<br><br>Vital signs (blood pressure and heart rate, and the rate of adverse events.<br><br>Patients' impressions from free description-type questionnaire.	Hospital	Compared the effects on quality of life, vital signs, and sleep quality between aromatherapy and conventional therapy during perioperative periods of the patients in this study.
56	Tarrasch et al., 2018	Israel	Breast cancer during adjuvant radiation therapy	58	Reflexology	**Multidimensional Quality of Life Scale-Cancer questionnaire (MQOLS-CA).**<br><br>Lee Fatigue Scale.<br><br>General Sleep Disturbance Scale	Cancer Center of a Medical Center	Evaluate the effect of reflexology on fatigue, pain, and quality of life in women with breast cancer during adjuvant radiation therapy.

						Numeric rating scale of pain intensity and pain visual analogue scale.		
57	Topcu et al., 2020	Denmark	Asthma	86	Reflexology	**Asthma quality of life questionnaire (AQLQ)**<br><br>Asthma control questionnaire (ACQ)<br><br>**EuroQol-5D (EQ-5D)**	Outpatient clinic	Assess the effect of reflexology and individualised homeopathy as an adjuvant treatment in asthma.
58	Uysal et al., 2017	Turkey	Colorectal cancer	60	Foot massage and reflexology	Introductory information form.<br><br>Common terminology criteria for adverse events.<br><br>**European Organization for Research and Treatment of Cancer Quality of Life Questionnaires (EORTC QLQ) C30 and CR29**	Department of Radiation Oncology of an oncology training and research hospital	Analyse the effect of foot massage administered using classical massage techniques and reflexology on the symptoms of people with colorectal cancer who receive chemoradiotherapy.
59	Vallim et al., 2019	Brazil	Breast cancer	54	Auricular acupressure	**European Organisation for Research and Treatment of Cancer Quality of Life Questionnaire Core 30 (QLQ-C30) – version 3.0**<br><br>**European Organisation for Research and Treatment of Cancer Quality of Life Questionnaire Breast Cancer 23 (QLQ-BR23)**	Oncology unit	Evaluate the effects of an auricular acupressure intervention on the quality of life of women with breast cancer undergoing chemotherapy treatment compared to those who did not undergo the intervention.

60	Wang et al., 2014	China	Diabetic with chronic kidney disease	62	Auricular acupressure	**Kidney Disease and Quality of Life Short-Form,**<br><br>**Short-form 36-item health survey questionnaire (SF-36).**<br><br>Examination of glycosylated hemoglobin (HbA1c) and estimated glomerular filtration rate.	Nephrology department in the medical college affiliated hospital	Assess the effectiveness of auricular acupressure for quality if life improvement in these patients.
61	Wilkinson et al., 2007	UK	Cancer	288	Aromatherapy -massage	State Anxiety Inventory (SAI).<br><br>Center for Epidemiological Studies Depression (CES-D) Scale.<br><br>**European Organisation for Research and Treatment of Cancer (EORTC) QLQ-C30 (version 3).**<br><br>Change in anxiety and/or depression between full case and borderline and non-case, or between borderline and non-case at 10 weeks post randomization.	Cancer care setting	Determine whether a course of aromatherapy massage confers greater improvement in clinically important anxiety and/or depression than does usual supportive care.<br><br>Examine whether aromatherapy massage produced greater improvement on self-reported anxiety, depression, pain, fatigue, nausea and vomiting, and global quality of life.

62	Wilkinson et al., 1999	UK	Palliative care	103	Aromatherapy -massage	**Rotterdam Symptom Checklist**.<br><br>State Anxiety Inventory (SAI-13.<br><br>Trait Anxiety Inventory (TAI-13.<br><br>Semi-structured questionnaire.	Palliative care centre	Evaluate: 1) the effectiveness of massage in improving the quality of life of patients with advanced cancer;<br>2) the effectiveness of aromatherapy massage in improving the quality of life of patients with advanced cancer; 3) patients' perceptions of massage.
63	Wyatt et al., 2017	USA	Breast cancer	256 (patient -carer dyad)	Reflexology	Demographic information (age, race, ethnicity, level of education, employment, income, and relationship to the caregiver).<br><br>Bayliss tool.<br><br>Data on cancer (stage, recurrence, metastasis, & medical treatments administered during the study)<br><br>**M.D. Anderson Symptom Inventory.**	Community based oncology clinics & cancer centres	Determine the effects of a four-week, homebased reflexology intervention delivered by a friend/family caregiver compared with attention control on: 1) patient symptom severity and interference with daily activities. 2) functioning and satisfaction with life. 3) perceived social support and quality of patient-caregiver relationship

						Patient-Reported Outcomes Measurement Information System (PROMIS) Tools.<br><br>**Quality of Life Index.**<br><br>**Multidimensional Scale of Perceived Social Support, Quality of Relationship Tool.**		To determine if the effects of the reflexology intervention on symptoms, functioning, and satisfaction with life are mediated by the perceived social support and quality of relationship with the caregiver.
64	Wyatt et al., 2012	USA	Breast cancer receiving chemo and/or hormonal therapy	385	Reflexology	**Physical function subscale - Short-form 36-item health survey questionnaire (SF-36).**<br><br>**Functional Assessment of Cancer Therapy–Breast (FACT-B).**<br><br>Brief Fatigue Inventory.<br><br>Brief Pain Inventory-Short Form. Center of Epidemiologic Studies–Depression (CES-D).<br><br>State-Trait Anxiety Inventory (STAI).	Community based medical oncology clin-ics	Test a consistent reflexology protocol with adequate sample sizes.<br><br>To evaluate the safety and efficacy of reflexology, a complementary therapy that applies pressure to specific areas of the feet.
65	Xu & Mi, 2017	China	Migraine with aura	98	Acupressure	Pain numeric rating scale.<br><br>Frequency of migraine.	Hospital	Explore the efficacy and safety of acupressure as an

						Times of using analgesics.<br><br>**Short-form 36-item health survey questionnaire (SF-36).**		adjunctive therapy to sodium valproate for relieving pain in patients with chronic migraine with aura.
66	Yeh et al., 2015	Taiwan	Primary hypertension	150	Auricular acupressure	Demographic characteristics (age, sex, education levels, marital status, employment, physical activity, smoking history, drinking history, body mass index, and waist-hip ratio).<br><br>Disease conditions (hypertension duration, number of antihypertensive drugs, other cardiovascular dis-eases, hypercholesterolemia, anti-cholesterol drugs, other medications and drug compliance).<br><br>Physiological parameters (Heart rate variability, High frequency, Low frequency, Low frequency/High frequency ratio, blood pressure, and heart rate.<br><br>**Short-form 36-item health survey questionnaire (SF-36).**<br><br>The 21-item Beck Anxiety Inventory (BAI-21).	Medical teaching hospital	Evaluate the effect of auricular acupressure on heart rate variability and quality of life in patients with primary hypertension.

Bold: Multi-domain tool included in search 2

Supplementary material: Table of included papers search 2

	Reference	Country	Population	Sample Size	Outcome measure(s)	Setting	Aim
1	Cella et al., 1993	USA	Cancer<br><br>Breast (33.3%),<br>Lung (33.3%),<br>Colorectal (33.3%)	45	FACT-G	*unclear*	Developed and validate a brief, yet sensitive, 33-item general cancer quality-of-life measure for evaluating patients receiving cancer treatment, called the Functional Assessment of Cancer Therapy (FACT) scale
2	Chang et al., 2000	USA	Cancer<br><br>Head and Neck (4.7%),<br>Lung (21.9%),<br>Gastrointestinal (13.3%),<br>Genitourinary cancer (37.8%),<br>Hematologic (16.7%) & Miscellaneous (5.6%)	233	ESAS	Medical oncology clinic outpatients and inpatient service	Describe the validation of the ESAS in a population of cancer patients who concurrently completed two other instruments, the Memorial Symptom Assessment Scale (MSAS) and the Functional Assessment of Cancer Therapy (FACT)
3	Easson et al., 2007	Canada	Advanced intra-abdominal malignancy<br><br>Ovarian cancer (41%),<br>Benign liver disease (14%),	61	ESAS<br><br>ESAS–Ascites Modification	Hospital outpatients	Assess the ability of existing symptom and quality-of-life questionnaires to detect change in symptoms after paracentesis

			Pancreatic cancer (9%),<br>Colorectal cancer (9%),<br>Breast cancer (9%),<br>Liver cancer (7%),<br>Unknown primary cancer (7%),<br>Endometrium (2%),<br>Mesothelioma (2%)		Memorial Symptom Assessment Scale Short Form<br><br>EORTC QLQ-C30<br><br>EORTC QLQ-PAN26		
4	Gao et al., 2012	UK	Specialist palliative care, cancer<br><br>Most common;<br>Lung cancer (20.7%)	714	GHQ-12	Cancer outpatient, general community, and palliative care settings	Determine the factor structure of the 12-item General Health Questionnaire (GHQ-12) across the cancer trajectory represented by samples from three cancer care settings and to appraise the item misfit and differential item functioning of the GHQ-12
5	Gough et al., 2019	USA	Advanced soft tissue sarcoma, cancer<br><br>Lung (67%),<br>Liver (21%),<br>Bone (15%),<br>Soft tissue (44%),<br>Lymph nodes (18%),<br>Other organ (6%)	66	EORTC QLQ-C30<br><br>Interview	Hospital	Explore qualitatively the individual constituents of HRQoL in two groups of patients with advanced STS: one group commencing first line Palliative chemotherapy, the other undergoing a period of 'active surveillance' having completed,

							and favourably responded to, first line chemotherapy achieving at least stable disease.<br><br>Examine how overall HRQoL and its components changed over time in both groups using the EORTC QLQ-C30 and qualitative methodologies, to explore in what ways palliative chemotherapy was valuable in terms of HRQoL
6	Gupta et al., 2008	USA	Cancer<br><br>Breast Cancer (26.3%), Colorectal (18.6%), Lung (16.4%), Prostate (4.5%), Pancreas (5.8), Other (28.5%)	954	Ferrans and Powers Quality of Life Index<br><br>EORTC QLQ C30	Cancer Treatment Centers of America at the Midwestern Regional Medical Center	Compare the European Organisation for the Research and Treatment of Cancer Quality of Life Questionnaire (QLQ-C30) and the Ferrans and Powers Quality of Life Index
7	Holden et al., 2019	USA, Canada	Parkinsonian disorders	210	Parkinson's disease Questionnaire-39<br><br>Patient-reported outcomes measurement information system (PROMIS-29)	Outpatient neuro-palliative care clinics	Describe the psychometric utility of four commonly used QOL scales in a population of PD patients receiving palliative care and define and compare the minimal clinically important differences and responsiveness to change for QOL scales in this population

					Quality of life in Alzheimer's disease scale<br><br>McGill QOL		
8	Le Fevre et al., 1999	UK	Palliative Care	79	Hospital Anxiety and Depression Scale<br><br>GHQ-12<br><br>Revised Clinical Interview Schedule	Palliative care inpatient setting	Compare the performance of the Hospital Anxiety and Depression Scale (HADS) with the 12-item General Health Questionnaire (GHQ-12) as a screening instrument for the detection of psychiatric disorders in a palliative care inpatient setting
9	Luo et al 2005	Singapore	Cancer<br><br>Breast cancer (40%),<br>Colorectal cancer (16%),<br>Leukaemia (11%),<br>Lung (9%),<br>Lymphoma (7%),<br>Germ cell tumour (5%),<br>Other cancer (11%)	57	EORTC QLQ-C30	Tertiary referral hospital	Examine the validity and reliability of the QLQ-C30 in English speaking Singaporean cancer patients
10	Novak et al., 2001	USA	Terminal Illness	38 (patient - carer dyads)	Hospice Comfort Questionnaire phase 1 (6-point scale)	Hospice	Test several formats of end-of-life comfort instruments for patients and closely involved caregivers

					Hospice Comfort Questionnaire phase 2 (4-point scale)<br><br>Total Comfort Line phase 1 (vertical)<br><br>Total Comfort line phase 2 (Horizontal)		
11	Philip et al., 1998	Australia	Cancer	80	ESAS<br><br>Rotterdam Symptom Checklist<br><br>Brief Pain Inventory	Palliative care services	Validate the modified Edmonton Symptom Assessment Scale against the two self-administered questionnaire commonly used in cancer care: the Rotterdam Symptom Checklist and the Brief Pain Inventory
12	Pratheepa wanit, Salek & Finlay, 1999	UK	Advanced Cancer receiving palliative care	52	McGill QOL<br><br>Patient Evaluated Problem Score	Hospital outpatient clinics and palliative care day centre	Assess the type of information given by patients in response to the two different techniques and compare the acceptability and value of these questionnaires in routine clinical assessment
13	Sherman et al., 2006	USA	Advanced cancer and Acquired Immune Deficiency Syndrome (AIDs)	101	McGill QOL<br><br>Quality of Life Scale - Family Version	AIDS clinic, private oncology practices, or inpatient settings	Establish the reliability of selected multidimensional quality of life instruments for both patients with cancer and patients with AIDS and cancer and AIDS caregivers.

							Identify differences in quality of life of patients with advanced cancer and AIDS, and their family caregivers with consideration of mortality, attrition, and compliance rates for patients with advanced illness<br><br>Examine differences in demographic variables between patients with cancer and AIDS and their family caregivers, and their potential influence as confounding variables when measuring quality of life
14	Stromgren et al., 2002	Netherlands	Advanced cancer	171	EORTC QLQ-C30<br><br>ESAS<br><br>Palliative Care Outcome Scale<br><br>McGill QOL<br><br>Memorial Symptom Assessment Scale	Department of Palliative Medicine at a Hospital	Map the ‘symptoms’ of patients referred to a department of palliative medicine, and to compare the resulting list of symptoms with five well-known questionnaires used to evaluate palliative care, in order to examine the extent to which the palliative needs described in the records can be measured by means of the questionnaires

15	Thekkumpurath et al., 2009	UK	Advanced cancer	150	Distress Thermometer<br><br>Brief Symptom Inventory-18<br><br>GHQ-12	Palliative care sites	Test the efficacy of three screening questionnaires in detecting all forms of psychological disorders (conceptualized as distress) in palliative care patients, most of whom had advanced cancer
16	Watanabe et al., 2012	Canada, Switzerland	Advanced cancer	Study 2: 20<br><br>Study 3: 160	ESAS<br><br>ESAS-r	Study 2: Tertiary cancer center<br><br>Study 3: Palliative care sites	Provide a synthesis of a program of research focusing on the psychometric properties of the ESAS
